# A novel, rapid, and practical prognostic model for sepsis patients based on dysregulated immune cell lactylation

**DOI:** 10.3389/fimmu.2025.1625311

**Published:** 2025-06-19

**Authors:** Chang Li, Mei He, PeiChi Shi, Lu Yao, XiangZhi Fang, XueFeng Li, QiLan Li, XiaoBo Yang, JiQian Xu, You Shang

**Affiliations:** ^1^ Department of Critical Care Medicine, Union Hospital, Tongji Medical College, Huazhong University of Science and Technology, Wuhan, China; ^2^ Hubei JiangXia Laboratory, Wuhan, Hubei, China

**Keywords:** sepsis, metabolism, epigenetic modifications, lactylation, early prognostic prediction, PPP1R15A

## Abstract

**Background:**

Sepsis is a global health burden characterized by high heterogeneity and uncontrolled immune response, with a notable lack of reliable methods for early prognosis and risk stratification. Epigenetic modifications, particularly lactylation, have recently emerged as key regulators in the early pathophysiology of sepsis. However, their potential for immune-related mortality risk stratification remains largely unexplored. This study aimed to investigate dynamic changes in lactylation during sepsis progression and to develop a rapid, lactylation-based prognostic signature.

**Methods:**

Blood transcriptional profiles and single-cell RNA sequencing data from septic patients were analyzed to assess glycolytic activity and lactylation in relation to patient mortality. Patients were stratified into subgroups using k-means clustering based on lactylation levels. Machine learning algorithms, integrated with pseudotime trajectory reconstruction, were employed to map the temporal dynamics of lactylation. A prognostic model was then constructed using lactylation-associated hub genes and validated in external transcriptomic datasets, a prospective single-center clinical cohort. The underlying mechanism was further explored *in vitro* using human monocytes.

**Results:**

The study systematically characterized the dynamic alterations in lactylation patterns and immune microenvironment across distinct patient clusters. A lactylation-based prognostic model was developed, comprising eight key genes (CD160, HELB, ING4, PIP5K1C, SRPRA, CDCA7, FAM3A, PPP1R15A), and demonstrated strong predictive performance for sepsis outcomes (AUC = 0.78 in the training cohort; AUC = 0.73 in the validation cohort). Temporal expression patterns of lactylation-related hub genes revealed dynamic immune responses throughout disease progression. In the prospective cohort of septic patients (N = 51), the model showed high predictive accuracy for survival, with AUCs of 0.82 (7-day), 0.80 (14-day), and 0.86 (28-day). Additionally, global lactylation levels were significantly elevated in THP-1 cells following treatment with Sephin1, a selective PPP1R15A inhibitor, suggesting a mechanistic link.

**Conclusions:**

Lactylation is significantly associated with increased mortality risk in sepsis. The proposed individualized prognostic model, based on dysregulated immune cell metabolism, accurately predicts early mortality and may inform optimized clinical management of septic patients.

## 1 Introduction

Sepsis is a critical condition involving organ dysfunction due to an abnormal host response to infection, marked by considerable variability and immune system imbalance (1). As a major global health challenge, sepsis has been recognized by the World Health Organization as a global health priority in 2017 due to its substantial acute mortality and long-term morbidity (2, 3). Thus, deeply comprehending the inherent pathophysiological changes in sepsis and the evolving immune responses during its onset is crucial for treating and managing sepsis patients (4). Regarding the clinical practice, due to the lack of sufficient and effective clinical parameters and molecular biomarkers, the rapid and reliable sepsis diagnosis/prognosis have long remained a significant challenge (5–7). However, current therapeutic strategies have failed to significantly reduce mortality rates in sepsis patients, highlighting the urgent need for more personalized treatment and deeper understanding of pathophysiology (8, 9).

Epigenetic modifications during sepsis could occur in the very early stage. As a novel epigenetic modification, lactylation has been shown to function in various diseases, including infection and cancer (10). As a product of the Warburg effect, high concentrations of lactate is the primary driver of lactylation (11). Similar to other post-translational modifications, lactylation plays diverse roles in different diseases (12). Lactylation mediates the immunosuppressive function of myeloid cells in tumor microenvironment, thereby facilitating tumor immune escape (13). In sepsis studies, researchers found that lactylation could increase the release of CIRP in macrophages, which triggers the PANoptosis in endothelial cells (14). Additionally, some researchers suggest that lactylation can also promote the macrophage release of exosomes containing HMGB1, which similarly causes damage to endothelial cells (15).

However, the effects mediated by lactylation in sepsis have not yet been fully elucidated. In recent years, emerging evidence suggests the lactate, a common metabolite of immune cells, could serve as a biomarker associated with clinical outcome in sepsis patients (12). However, its utility is limited in clinical practice due to several reasons. For example, the level of lactate shows non-specific elevation across multiple diseases. Moreover, as a result of measurement limitations, the current clinical detection could only capture the circulating lactate. This may consequently lead to the decreased sensitivity in the prognosis of sepsis patients, since the immune cells exhibit intracellular lactate accumulation during the early phase of sepsis due to the metabolic dysfunction (16).

In our current study, we developed a novel approach to estimate the dysregulated metabolism of immune cells. We integrated lactylation-related genes previously published (17–19), and conducted a comprehensive analysis on the whole blood transcriptional profiling of sepsis patients using machine learning and pseudotime analyses. The hub genes were explored and a prognostic model was constructed, which showed a stable and good performance in the prognosis of sepsis patients. Moreover, we collected the PBMC samples from clinical sepsis patients in our own hospital to further validate our findings, and the results demonstrated a high accuracy and good performance. The potential underlying mechanism has been explored through *vitro* experiments. The lactylation and its related molecules demonstrate significant potential as a sepsis prognostic marker.

## 2 Materials and methods

### 2.1 Data preprocessing

The Gene Expression Omnibus (http://www.ncbi.nlm.nih.gov/geo/) provided genome-wide blood transcriptional profiles for both sepsis patients and healthy individuals, incorporating the GSE65682 cohort (N= 802) and GSE95233 cohort (N=124). The detailed information of the cohorts is included in **Supplementary Table S1**. The data preprocessing was executed following these steps: 1). Exclude samples that lack clinical information. 2). Log2-transformation of the Data. 3). Retention the genes of expression >0.

For scRNA-seq data of human PBMCs (survivor and non-survivor of gram-negative sepsis patients, N=12), preprocessing was performed using Seurat (v 5.0.1). Specifically, the transcriptional profiling was filtered with UMI count >1000, gene features between 200~6000, and mitochondrial percentage < 25%. Then the filtered gene matrix were normalized through LogNormalize method in seurat with default parameters. After principal component analysis (PCA) was performed, dimensional reduction was achieved via t-SNE. Cell clusters were identified at a resolution of 1.0. Then we used marker genes and “FindAllMarkers” function to define each cell cluster, for example, Monocyte (CD14), T cells (CD3), DCs (FCER1A), B cells (MS4A1) (Details in **Supplementary Table S2**). The processed transcriptional profiles were utilized for subsequent analysis.

### 2.2 Gene set enrichment analysis

We employed GSEA to delineate differentially enriched gene sets in sepsis patients compared to healthy individuals. Additionally, GSVA was performed to calculate the gene sets (including lactylation and immune cells) enrichment, which allows for the assessment of biological activity levels in a single sample.

### 2.3 DEG analysis and survival analysis

DEG were identified by “limma” package in R, and FDR <0.05 was set as the threshold. Kaplan-Meier survival analysis coupled with Log-rank testing was performed to determine significant differences in mortality rates between groups.

### 2.4 Identification of distinct sub-clusters in sepsis patients

The lactylation-related sub-clusters in sepsis patients were identified using k-means consensus clustering (“ConsensusClusterPlus” package in R) based on the expression profile of the lactylation-related genes. To ensure the stability of the classification, the calculation was repeated 1,000 times iteratively. And the knee point was utilized to determine the optimal cluster number.

### 2.5 Calculation of immune cell abundance and microenvironment activity in sepsis patients

Using established immune cell gene signatures, we performed GSVA to quantify immune cell infiltration patterns. To minimize potential biases associated with relying on a single computational method, we further validated the reliability of the results by comparing them with those obtained from alternative immune cell abundance estimation methods, including EPIC and MCP-counter. Furthermore, The ESTIMATE method was employed to assess immune infiltration levels across all samples, providing an overall assessment of the immune microenvironment. Additionally, we applied the Immunophenoscore (IPS) algorithm to calculate detailed information, including EC (effector cells), CP (immune checkpoints), MHC (MHC molecules), SC (suppressor cells), and total Immunophenoscore.

### 2.6 Construction of lactylation-related prognosis signature

The sepsis patients sample in GSE65682 cohort were used to construct the lactylation-related prognosis signature. The hub gene filtering procedure was conducted according to the following steps: 1) Identification of DEGs: We conducted two parallel differential expression analyses: between Cluster 3 versus other clusters, and between deceased versus surviving patients. 2) The intersecting genes were subjected to univariate Cox proportional hazards regression analysis, and p<0.05 was set as the threshold. 3).LASSO regression and Elastic Net algorithms were applied to reduce the number of variables and avoid overfitting 4). Multivariate Cox proportional hazards regression to assess their prognostic significance. P value <0.05 were considered statistically significant and included to construct the lactylation-related prognosis signature.

Patients were randomly allocated to training (70%) and validation (30%) cohorts using a stratified randomization approach. Multivariate Cox regression analysis was performed in the training set, and the regression coefficients were utilized to construct the lactylation-related prognosis signature. ROC curves and AUC values were used to evaluate the performance of signature, and the validation set was used to confirm the results.

### 2.7 Pseudotime analysis

Pseudotime analysis is a widely used method in transcriptomics that infers the temporal sequence of cellular development or changes by ordering transcriptomic data along a specific biological trajectories. The pseudotime analysis was performed using “Monocle2” package in R with default parameters.

### 2.8 Western blot

After treatment, cells were harvested, and total protein was extracted. The proteins were transferred onto a PVDF (polyvinylidene difluoride) membrane after SDS-PAGE gels electrophoresis. The PVDF membrane was first incubated with primary antibodies for 12 hours, followed by incubation with HRP-conjugated secondary antibodies for 1–2 h at room temperature. The protein bands were detected using a Bio-Rad detection system with ECL solution kit. Primary antibodies used in our study were listed as follows: PPP1R15A (Abclone, #A16260, 1:1000), AARS1 (Abclone, #A15017, 1:2000), AARS2 (Proteintech, #22696-1-AP, 1:1500), LDHA (Abclone, #A1146, 1:500), GLUT1 (Abclone, #A11208, 1:500), pan-Klac (PTMBio, #1401RM), Na+/K+-ATPase (Abclone, #A11683, 1:1000). The HRP-conjugated anti-mouse and anti-rabbit secondary antibodies were purchased from Abclone.

### 2.9 PBMC isolation and real-time quantitative PCR analysis

Within the first 24h of ICU admission, blood samples (2.5ml) were collected from patients. The peripheral blood mononuclear cells (PBMCs) were isolated within 1 hour using Ficoll (Sigma-Aldrich, USA) density gradient centrifugation according to the manufacturer’s protocol.

Cells were harvested, and total RNA was extracted using TRIzol (Biosharp, #BS258A) according to the manufacturer’s instructions. Then the extracted RNA was reverse-transcribed into cDNAs using reverse transcription kit, and the qPCR reaction was performed using the SYBR Green mix in the Bio-rad real-time PCR system. The reverse transcription kit and the SYBR Green mix were purchased from Yeasen Bio. The primer sequences are listed in **Supplementary Table S3**.

### 2.10 Statistical analysis

Bioinformatic analysis was conducted in R (v4.3). The whole blood transcriptional profiling of sepsis patients cohorts were derived from GEO database. DEG analysis was conducted using the “limma” package. LASSO regression and Elastic Net algorithms were utilized to reduce the number of variables. Univariate and multivariate Cox regression analyses were conducted to filter genes. Kaplan-Meier (KM) plots was used to show the survival difference between different groups, and Log-rank test was used to determine the statistical significance. Pearson correlation analysis was performed to evaluate the relationships between continuous variables. For experimental data, ImageJ and Graphpad Prism were used to complete statistical analysis. The statistical difference between two groups was determined using unpaired Student’s t-test. P<0.05 was considered statistical significant in our study (*: p< 0.05, **: p < 0.01, ***: p < 0.001).

## 3 Result

### 3.1 Classification of lactylation-related sub-clusters in sepsis patient

**Figure 1:**
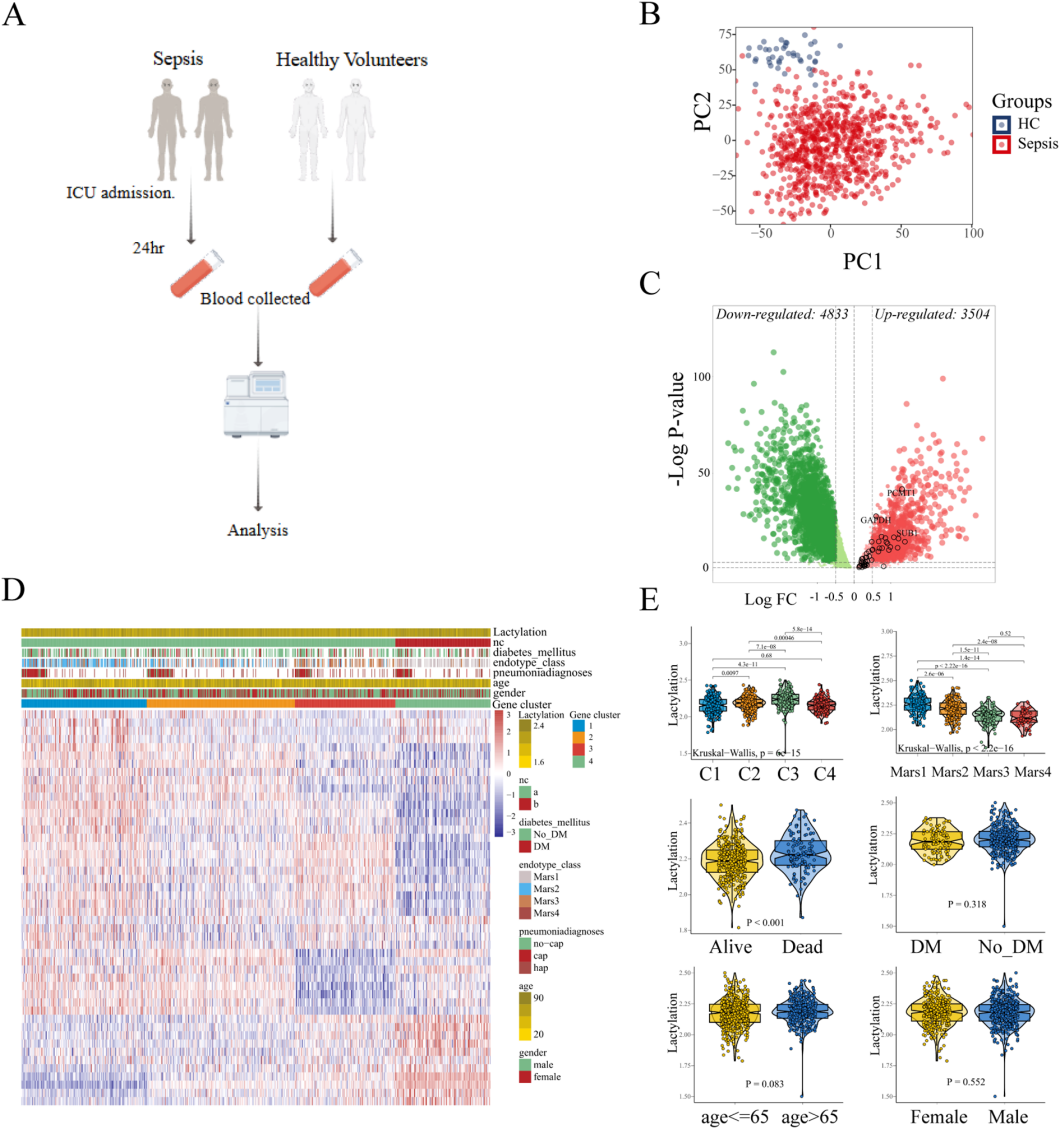
Identification of the lactylation-based sub-clusters in sepsis. **(A)** Expression data of 760 sepsis patients and 42 healthy volunteers. **(B)** Principle components analysis (PCA) shows the distinct blood transcriptional profiling between healthy volunteers and sepsis patients. **(C)** The differentially expressed genes are displayed in the volcano plot, with 3,504 up-regulated and 4,833 down-regulated genes identified. The black circles represent the expression changes of lactylation-related genes. **(D)** Sepsis patients were divided into four sub-clusters according to the consensus clustering. **(E)** The comparison of lactylation based on different clinical characteristics.

Sepsis is a disorder with significant heterogeneity. To explore the molecule features in sepsis patient, we obtained the public whole blood transcriptional profiling from GSE65682 in GEO database, which contained the gene expression data of 760 sepsis patients and 42 healthy volunteers (**Figure 1A**). As shown in **Figure 1B**, the principal component analysis (PCA) showed the obvious difference transcriptional pattern between sepsis patients and healthy controls. Next, we performed GSEA to evaluate the biological function/process enriched in sepsis. Diverse hallmarks were enrich in sepsis, such as IFN-γ response, cholesterol homeostasis, and epithelial mesenchymal transition (EMT) (**Supplementary Figure S1A**). Notably, we found that glycolysis is significantly enriched in sepsis. The high-intensity glycolysis results in the high concentration of lactate, which is the primary drive of lactylation. Lactylation, a novel post-translational modification driven by lactate metabolism, exhibits multifaceted regulatory roles in cellular processes, including epigenetic reprogramming (e.g. competes with acetylation at shared lysine sites), metabolic adaptation, and immune modulation. These lactylation-induced effects prompt us to investigate their potential roles in the pathogenesis and progression of sepsis.

To characterize the lactylation level in each sample, we curated 332 lactylation-related genes from prior studies (19), of which 48 genes were upregulated in sepsis (**Figure 1C**). GSVA was used to compute the enrichment level of the lactylation in each samples with the lactylation-associated gene sets as a reference, which indicated the lactylation-associated genes transcriptional level, thereby indirectly reflecting the lactylation enrichment levels in the samples. Using consensus clustering (k-means) and GSVA with the 48-gene set, we identified four stable sub-clusters (optimal K = 4, determined by CDF; **Supplementary Figures S1B, C**). The enrichment level of lactylation in blood samples was inferred through the GSVA of lactylation-related gene set. Sepsis patients were stratified into four sub-clusters with distinct lactylation enrichment patterns (cluster 1-4, n = 197, 253, 166, 144, respectively. **Figure 1D**). Lactylation enrichment levels did not correlate with age, sex, diabetes history (p > 0.05; **Figure 1E**), or pneumonia type (CAP vs. HAP; **Supplementary Figure S1D**). However, patients with severe clinical status or mortality exhibited higher lactylation (**Supplementary Figure S1E**), and Cluster 3 (highest lactylation) was enriched for Mars I endotype patients—a subgroup with the worst outcomes. These findings suggest lactylation may drive poor prognosis in sepsis.

### 3.2 Evaluation of immune landscape across sub-clusters

**Figure 2:**
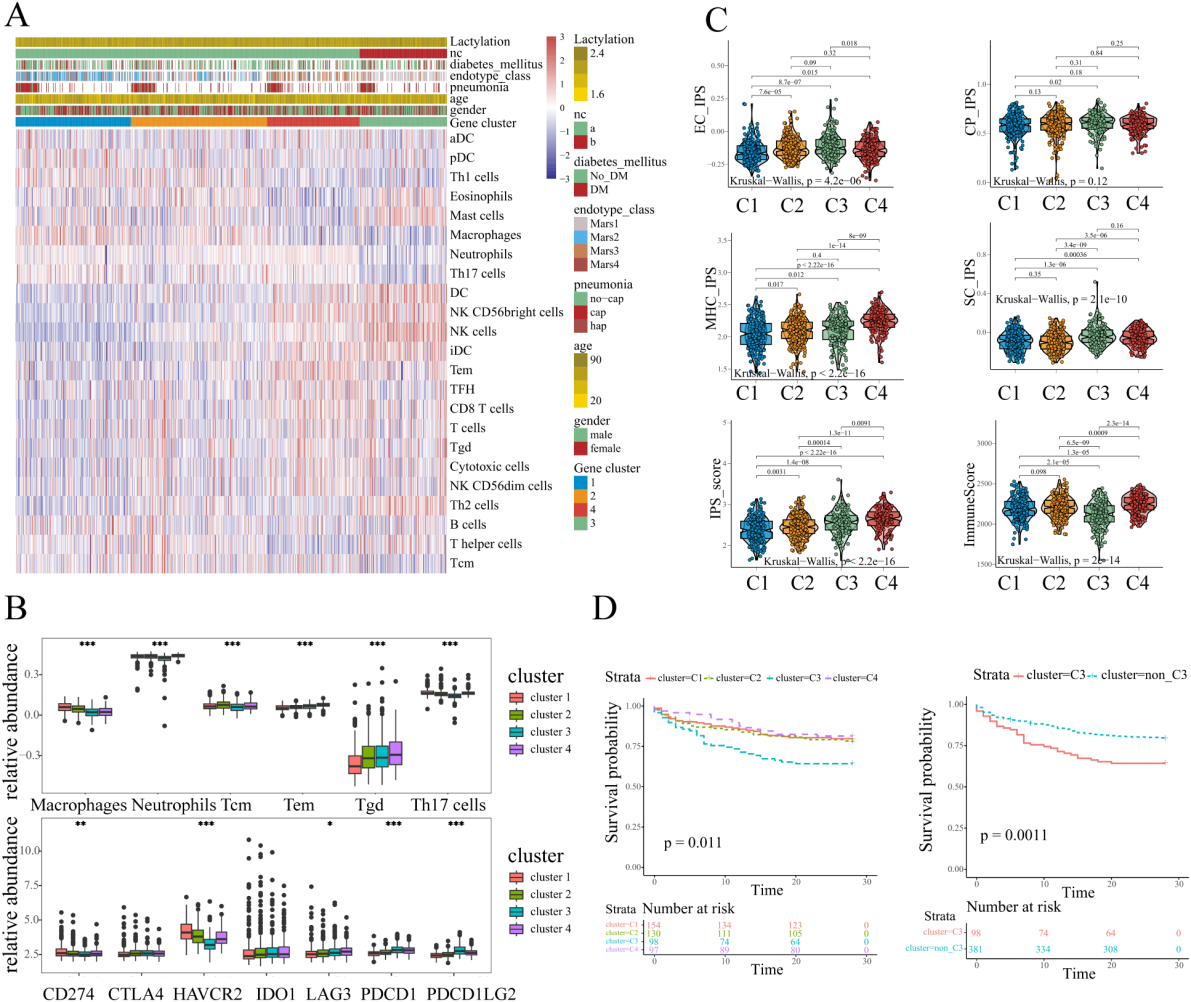
The immune characteristics of different sub-clusters. **(A)** The immune landscapes of different sub-clusters annotated with the lactylation enrichment level and clinical characteristics (history of diabetes mellitus, endotype, pneumonia, age, and gender). **(B)** The abundance of immune cells and immune checkpoints exhibited distinct patterns. One-way ANOVA was employed for statistical analysis among four sub-clusters: *p < 0.05, **p < 0.01, and ***p < 0.001. **(C)** The clusters3 showed lower immunoreactivity and higher immunosuppressive effects according to ESTIMATE and IPS scores. **(D)** Kaplan-Meier plot illustrated survival difference among sub-clusters, and patients in cluster3 had the poorest outcome (log-rank test, p<0.05).

To evaluate the immune landscape in each sub-cluster, ssGSEA was conducted to quantify immune cell abundance. Considering the potential biases brought by a single calculation, two additional algorithms EPIC and MCP-counter were utilized to infer the immune states. The abundance of the same type of immune cells calculated by different methods were compared (**Supplementary Figure S2**). According to the correlation analysis, the 6 overlapping immune cells (B cells, CD8+ T cells, total T cells, DCs, Neutrophils, Monocyte/Macrophage) showed a high degree of similarity with the relative abundance calculated by ssGSEA. For example, the relative abundance of CD8+ T cells calculated by ssGSEA was consistent with the other two quantification methods (EPIC: 0.57. MCP-counter: 0.63, Pearson correlation analysis, **Supplementary Figure S2**), which confirmed the stability of the results. As depicted in the heatmap in **Figure 2A**, the patterns of immune cell abundance showed distinct differences among different sub-clusters. Specifically, cluster-1 was characterized by a high level of macrophages, neutrophils, and Th17 cells. cluster-2 tended to have a relatively higher abundance of central memory T cells (Tcm). cluster-4 exhibited higher infiltration of γδ Tcells and effector memory T cells (Tem). As for cluster-3, the DCs and NK cells showed an evident increase, while the abundance of other immune cells (macrophages, neutrophils, Th17 cells, CD8+T) appeared to be lower as compared to the other sub-clusters (**Figure 2B**).

Additionally, we evaluated the expression of immune checkpoints which might have potential function in infectious diseases (20). The results revealed that several immune checkpoints including PD1(PDCD1)/PDL1(CD274)/PDL2(PDCD1LG2), CTLA4, TIM3(HAVCR2), IDO1, and LAG3 showed different expression patterns among four sub-clusters. The results revealed the apparently high levels of PD1 and PDL2 expression in cluster-3 samples (**Figure 2B**). Moreover, the cluster-3 exhibited the lowest expression level of HAVCR2 as compared to the clusters 1, 2, and 4. The results indicated that the different sub-clusters showed distinct immune checkpoint expression and immune cell abundance patterns. To further analyze the immune states in each sub-cluster, IPS (Immunophenoscore) and ESTIMATE algorithms were conducted. In line with the above analysis, the IPS results showed that cluster-3 exhibited a relatively higher suppressor cells scores among four sub-clusters (**Figure 2C**), indicating the higher immunosuppressive effects. Moreover, the samples in cluster-3 appeared to have the lowest immune scores according to the ESTIMATE, which might indicate the low immunoreactivity in cluster-3 (**Figure 2C**). Despite exhibiting elevated IPS scores, cluster 4 demonstrates concurrently high suppressor cell (SC) scores and upregulated immune checkpoint molecule expression. Notably, the immune checkpoint HAVCR2 shows significant over-expression in clusters 1, 2, and 4 compared to cluster 3. These findings collectively indicate that potential immunosuppressive effects are not exclusive to cluster 3 and distinct immune dysregulation patterns exist across sepsis sub-clusters.

Considering the distinct immune landscape shown above, patients in cluster 3 displayed markedly elevated lactylation levels, and a distinct immune cells abundance pattern. Strikingly, sepsis patients in this sub-cluster exhibited upregulation of immune checkpoint molecules and pronounced immune activation/immunosuppressive state, collectively suggesting a profound state of immune dysregulation. Therefore we further explored the prognosis between cluster 3 and other clusters. The Kaplan-Meier curves illustrated the survival difference across different clusters, and the results were statistically significant (p=0.011, **Figure 2D**). Obviously, the sepsis patients in cluster 3 experienced the worst prognosis as compared to the other clusters (**Figure 2D**). Given the previous analyses, the patients in cluster-3 appeared to have a higher degree of lactylation, lower immunoreactivity, higher immunosuppressive effects, and a poor prognosis. These results collectively identify cluster 3 as a distinct lactylation-driven sub-cluster with poor prognosis, guiding our subsequent hub gene analysis.

### 3.3 Key genes related to lactylation in sepsis

**Figure 3:**
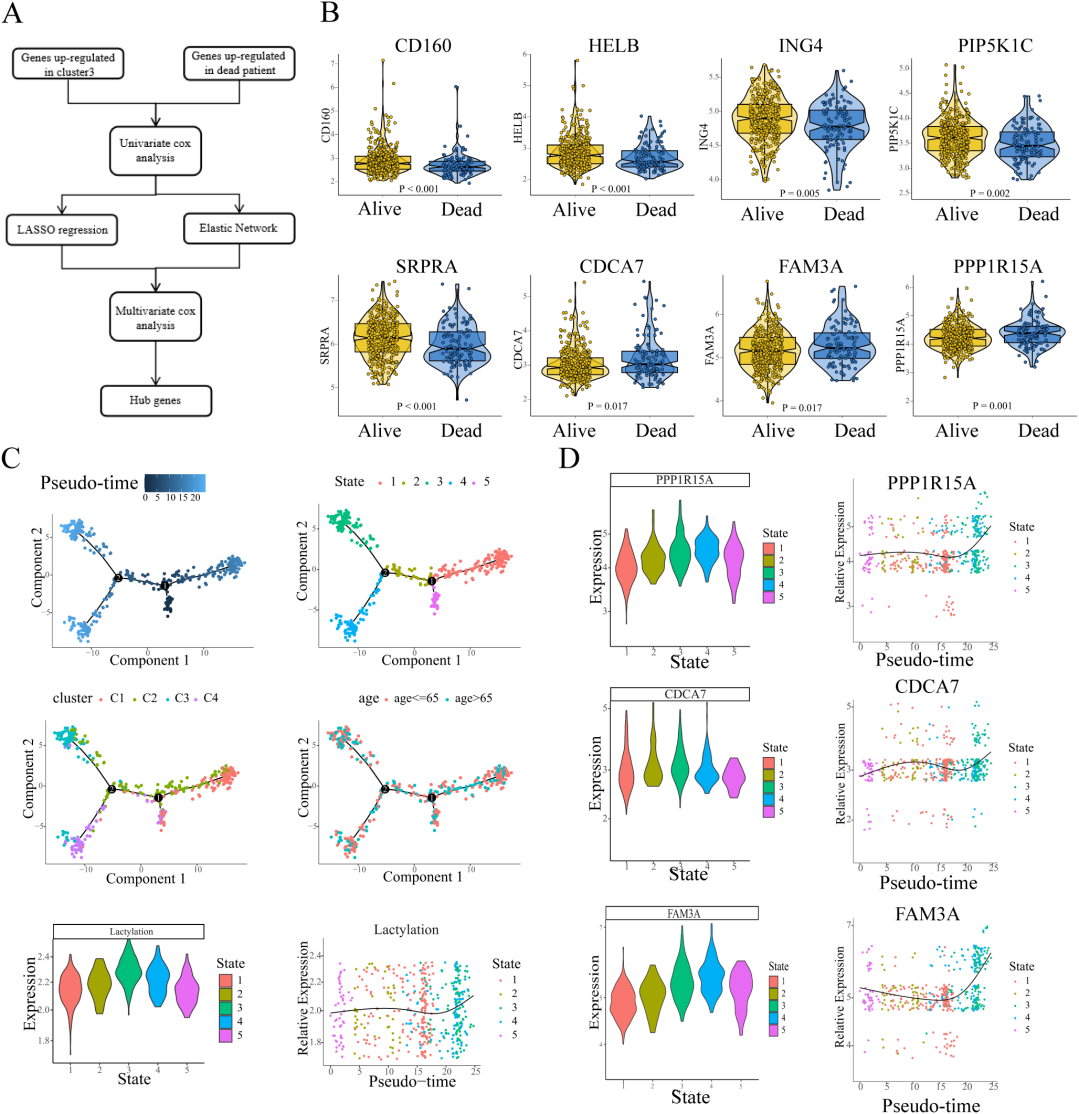
The detection and isolation of the key genes. **(A)** Flow chart showed the screening and selection of hub genes. **(B)** The expression of hub genes exhibits differential profiles between survivors and non-survivors. **(C)** Pseudo-time trajectories depicted the variation of lactylation in the sepsis process. **(D)** Pseudo-time trajectories showed the expression level of hub genes (HR>1) changed with the enrichment level of lactylation.

We sought to excavate the key genes based on the lactylation-related clusters, and a series of bioinformatic analyses and machine learning approaches were performed in sequence. The workflow is presented in **Figure 3A**. Gene selection pipeline proceeded as follows: First, we identified differentially expressed genes (DEGs) between cluster 3 and non-cluster 3 septic patients. Subsequently, we extracted DEGs between sepsis survivors and non-survivors. The intersection of these two steps yielded our candidate genes for further analysis. We next performed univariate Cox proportional hazards regression, and genes without HR statistical significance were filtered out. To reduce the number of candidate genes, we applied two machine learning approaches LASSO and Elastic Net. The LASSO algorithm, which imposing an L1 penalty on the regression, is widely employed in variable selection and identification of the most relevant biomarkers in scientific research. Similar to LASSO, the Elastic Net algorithm, combining the advantages of L1 penalty and Ridge regression (L2 penalty), has been commonly employed in robust variable selection. The variable selection process via LASSO regression and Elastic Net is presented in the supplementary figures (**Supplementary Figure S3A**). After the machine learning screening, Multivariate Cox proportional hazards modeling was employed to screen for genes exhibiting independent prognostic significance. Ultimately, 8 key genes were identified: CD160, HELB, ING4, PIP5K1C, SRPRA (HR < 1); and CDCA7, FAM3A, PPP1R15A (HR > 1), and the hub genes showed apparent differences in expression between alive and dead sepsis patients (**Figure 3B**).

To further explore the continuous process in sepsis, we applied pseudotime analysis to reconstruct the dynamics. The results revealed that all the developmental trajectory of sepsis were assigned into five states (**Figure 3C**, depicted in different colors) with two branch points. State 5 was selected as pseudotime root due to its minimal calculated lactylation level, representing the presumptive starting point of lactylation dynamics in sepsis progression. From State 5, the developmental trajectory crosses the branch point 1 and transitions to State 2, followed by a transition to State 3/4, while moving rightward leads to State 1 (**Figure 3C**). Overall, the leftward trajectory (state 5-2-3,4) exhibits a gradual increase in lactylation levels, whereas the rightward trajectory (to State 1) shows no significant increase (or a slight decrease). Temporally, State 1 slightly precedes State 3/4, and throughout the developmental process, lactylation levels tend to increase (**Figure 3C**). Notably, patients in cluster 3 are predominantly distributed at the terminal branch of State 3, while cluster 1 patients are primarily located at the end of state 1 branch. The pseudotime trajectory indicated that in the process of sepsis, some patients might develop or sustain a high level of lactylation, resulting in a high rate of mortality. Then we evaluated the expression of 3 hub genes (CDCA7, FAM3A, PPP1R15A, HR>1) in the trajectory process in sepsis (trajectory process of the other 5 hub genes were shown in **Supplementary Figure S4**). The results showed that all trajectories exhibited consistent upregulated tendencies. From the root state 5, all three hub genes showed lower expression compared to the other states. In line with the changes of lactylation, during the continuous process of sepsis, all three genes gradually increased, and reached the peak when the branch reached to the terminal state (**Figure 3D**). The expression of the three key genes appeared to reflect the levels of lactylation in relation to the results.

### 3.4 Construction of the lactylation-related prognostic signature

In previous studies, sepsis is a systemic inflammatory dysfunction with complicated changes, which lacks reliable prognostic biomarkers due to the individual heterogeneity. Considering the above results, we endeavored to establish a lactylation-related prognostic signature in the following analyses. First, all samples were randomly assigned into the training set (n=335) and the validation set (n=144) with a 7:3 ratio. The multivariate Cox regression analysis was performed, the 8-hub genes (CD160, HELB, ING4, PIP5K1C, SRPRA, CDCA7, FAM3A, PPP1R15A) and their calculated coefficients were utilized for the establishment of the lactylation-related prognostic signature. The regression coefficients table and hazard ratios (HR) of these 8 candidate genes are illustrated in the corresponding figures (**Supplementary Figure S3B**).

**Figure 4:**
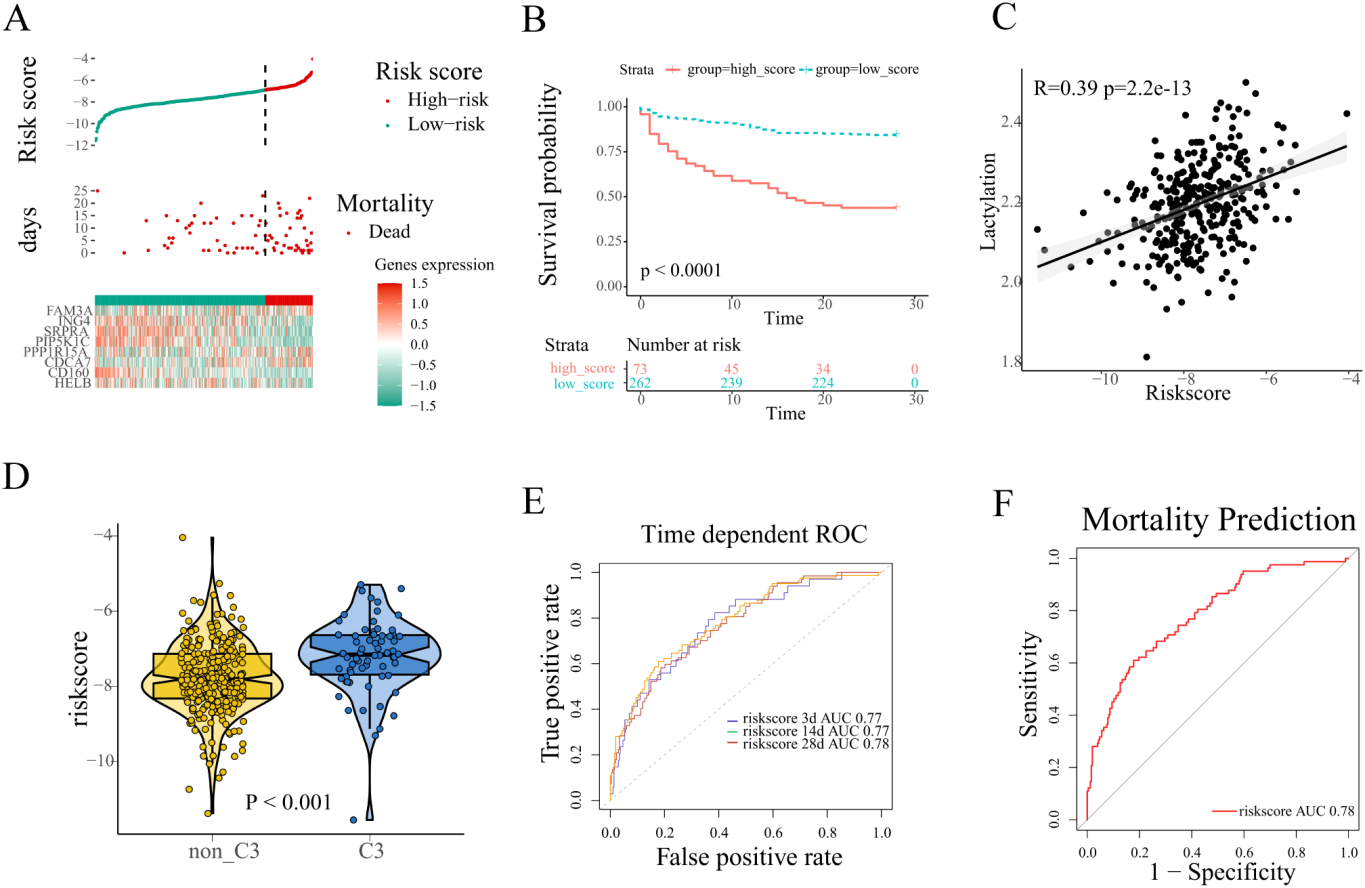
Construction of a lactylation-related prognosis signature. **(A)** The distribution of risk scores, survival time, expression patterns of signature genes in the training set. **(B)** Kaplan-Meier plot revealed evident survival outcome difference between high and low risk groups (log-rank test, p<0.001). **(C)** Pearson correlation analysis of the correlation between risk scores and lactylation. **(D)** The comparison of risk scores between different lactylation-related subclusters. **(E, F)** The performance of the signature was evaluated with ROC curves in the prediction of survival time and mortality of sepsis patients.

Sepsis patients were stratified into high and low risk groups based on the optimal cutoff value determined by X-tile software (**Figure 4A**). Patients stratified into the high-risk group exhibited poorer survival outcomes coupled with upregulated expression of prognostic risk genes compared to their low-risk counterparts (FAM3A, CDCA7, PPP1R15A, **Figure 4A**). Kaplan-Meier analysis revealed significantly worse survival outcomes in high-risk patients compared to low-risk counterparts (log-rank p<0.0001, **Figure 4B**). Furthermore, Pearson correlation analysis demonstrated a robust association between prognostic risk scores and lactylation levels (**Figure 4C**).Therefore we evaluate the risk score among all four sub-clusters, and patients in cluster-3 showed the highest (**Figure 4D**). The performance of the prognostic signature was further examined with ROC curves and the corresponding AUC values. In the training set, ROC analysis demonstrated strong predictive performance of the lactylation-related signature for survival time with AUCs 0.77 at 3 days, 0.77 at 14 days, and 0.78 at 28 days, respectively (**Figure 4E**). Notably, the signature was applied in the mortality prediction of sepsis patients, and the ROC curve indicated a good accuracy with 0.78 AUC value (**Figure 4F**).

In order to assess the stability of the lactylation-related prognostic signature, The prognostic signature’s predictive performance was rigorously validated in the validation cohort using the same risk score cutoff established in the training set. Consistent with training results, high-risk patients exhibited both elevated expression of risk genes (**Supplementary Figure S3C**) and significantly reduced survival times compared to low-risk counterparts. Survival analysis was conducted, and the result was depicted in the Kaplan-Meier plot. Similar to the training set, the significant survival difference was shown between the high and low risk group (Log-rank test, p<0.0001, **Supplementary Figure S3D**). The same positive correlation was observed between risk score and lactylation according to the Pearson correlation analysis in the validation set (**Supplementary Figure S3E**). In the prognostic prediction of patients survival time, the AUC value of the lactylation-related prognostic signature was 0.73 at 3 days, 0.70 at 14 days, 0.73 at 28 days, respectively (**Supplementary Figure S3F**). Additionally, the AUC value reached 0.73 in the prediction of mortality in the validation set (**Supplementary Figure S3G**). Taken together, the analyses establish that the lactylation-associated prognostic model maintains high accuracy and stability.

### 3.5 Dysregulated lactylation a common phenomenon in sepsis

**Figure 5:**
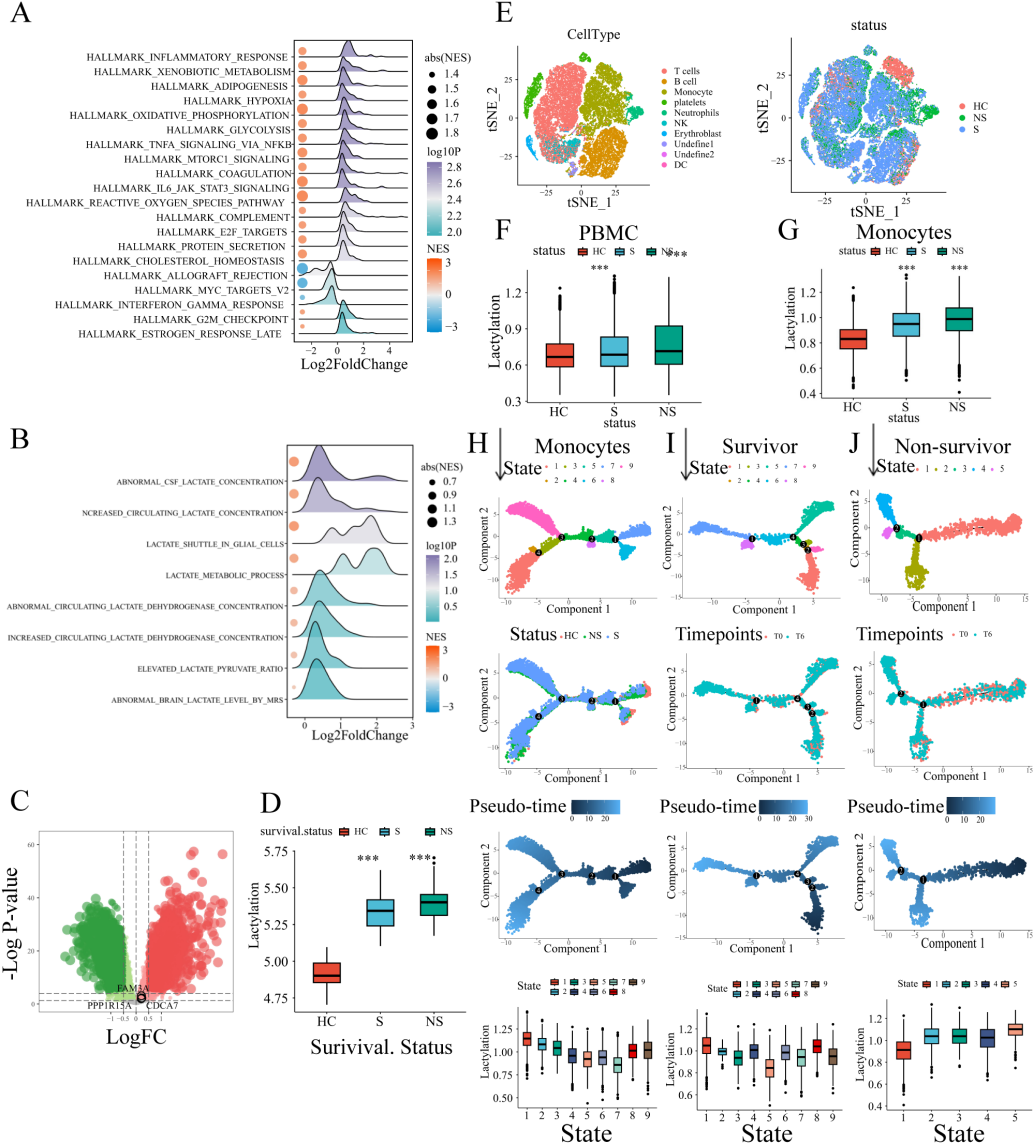
External validation of the lactylation-related signature. **(A, B)** GSEA analysis results of biological processes enriched in sepsis patients. **(C)** The risk genes (FAM3A, PPP1R15A, CDCA7) up-regulated in sepsis were depicted in the volcano plot. **(D)** Comparison of lactylation enrichment level between septic patients (non-survivors/survivors) and healthy controls (i.e. 1. Septic non-survivors vs healthy controls. 2.Septic survivors vs healthy controls). Statistical significance between groups (vs. healthy controls) is indicated above the bars: ***p < 0.001. **(E)** The t-sne plot of single-cell RNA sequencing data annotated with immune cells or sample groups. **(F)** The whole PBMC lactylation enrichment level was higher in sepsis patients. The comparison included septic non-survivors vs healthy controls and septic survivors vs healthy controls. Statistical significance (vs. healthy controls) is indicated above the bars: ***p < 0.001. **(G)** The monocyte lactylation enrichment level was elevated in sepsis patients. The comparison included septic non-survivors vs healthy controls and septic survivors vs healthy controls. Statistical significance (vs. healthy controls) is indicated above the bars: ***p < 0.001. **(H)** Pseudo-time trajectories of monocytes with status and states depicted in the plots. **(I)** Pseudo-time analysis of monocytes in survivor sepsis patients. **(J)** Pseudo-time analysis of monocytes in non-survivor sepsis patients.

For a comprehensive understanding of lactylation in sepsis, another whole blood transcriptional profiling was analyzed (GSE95233), which contained 51 septic shock patients and 22 healthy volunteers with corresponding clinical information. GSEA was performed, with hallmarks and several genesets related to lactate set as the references. In comparison to healthy volunteers, glycolysis and lactate-related pathways were significantly enriched in the sepsis patients (**Figures 5A, B**), which was consistent with the former results. The result indicated that the lactylation level increased in sepsis. The lactylation gene set was utilized to quantify the level of lactylation using GSVA. Similarly, the result revealed that the level of lactylation is obviously higher in sepsis patients as compared to the healthy volunteers. Additionally, in line with the former results, the three risk genes (FAM3A, PPP1R15A, CDCA7) were up-regulated in the sepsis patients (**Figure 5C**). Notably, the patients who did not survive depicted the highest quantification of lactylation (HC: healthy controls. S:survivors. NS:non-survivors, **Figure 5D**), indicating the correlation between poor prognosis and lactylation.

To investigate lactylation dynamics in sepsis, we analyzed single-cell RNA-seq data of human PBMCs from GEO (GSE167363), including healthy controls (n=2), survivors (n=6), and non-survivors (n=4). After quality control (**Supplementary Figure S5A**), dimension reduction, and clustering, the whole cells were assigned into different clusters. The canonical markers of immune cells were annotated in the t-sne plot (**Supplementary Figure S5B**). Based on the canonical markers and the SingleR cell type annotation tool, cell clusters have been defined, including T cells, platelets, B cells, monocytes, NK cells, neutrophils, DCs, and erythroblast (**Figure 5E**). GSVA revealed significantly elevated lactylation levels in sepsis patients (**Figure 5F**). Monocytes, the primary innate immune cells in PBMC, were separated for further analysis. Similarly, the lactylation level of monocytes was significant higher in sepsis patients, especially in patients who did not survive (**Figure 5G**). Subsequentially, the pseudotime analysis was conducted to evaluate the changes of lactylation level in monocytes during the process of sepsis. The whole monocytes were classified into eight states with four branch points (**Figure 5H**). As depicted in the trajectory, the healthy controls were located in the terminal of states 6/7, therefore the states 6/7 were determined as the root states (**Figure 5H**). Obviously, during the development of process (from rightward to the leftward in the trajectory plot), the monocytes exhibited a continuous increase of lactylation level (**Figure 5H**). Additionally, we analyzed the monocytes pseudo-time trajectory of sepsis survivors and sepsis non-survivors, respectively. The cells were assigned into different states, and the root states were determined based on the sample time-points referred by the clinical information (**Figures 5I, J**). In the survivor patients, the lactylation level of monocytes showed a slightly decrease tendency across the development process (**Figure 5I**). While in the non-survivor patients, the monocytes exhibited an evident increase in lactylation (**Figure 5J**). Combining the above results, the lactylation dysregulation is a common condition during the development of sepsis. The different alterations of lactylation between survivors and non-survivors indicated the potential association between lactylation and poor survival outcome.

### 3.6 Dynamic changes of lactylation and key genes during the progression of sepsis

**Figure 6:**
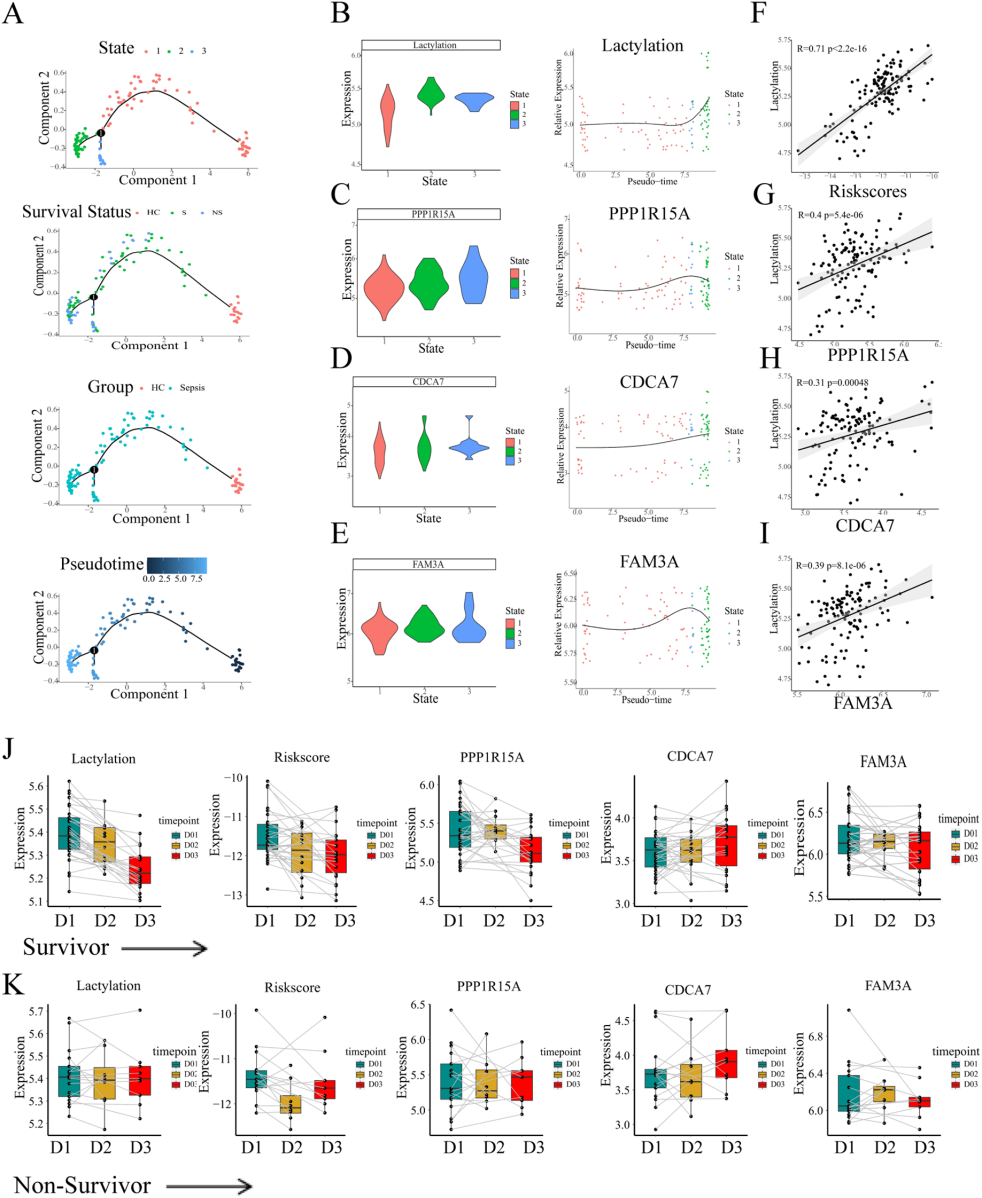
The continuous changes of lactylation and key genes in sepsis. **(A)** Pseudo-time analysis of blood transcriptional profiling in GSE95233 cohort. **(B-E)** The changes of lactylation and three risk genes (FAM3A, PPP1R15A, CDCA7) in temporal sequence according to the pseudo-time trajectories. **(F-I)** Pearson correlation analysis of the correlation between risk scores or risk genes and lactylation. **(J)** The continuous changes of lactylation, risk scores, and expression of risk genes in survivors. **(K)** The continuous changes of lactylation, risk scores, and expression of risk genes in non-survivors.

We further explored the changes of lactylation in the GSE95233 cohort. Pseudotime trajectory depicted the development of sepsis. The whole cohort samples were classified into three states with one branch point. According to the corresponding clinical information, the healthy controls (HC) were distributed mainly in the end of state 1. Therefore state 1 was defined as the root state (**Figure 6A**). Similar to the previous results, the lactylation level showed an obvious increase throughout the temporal sequence (**Figure 6B**). Furthermore, expression analysis of the three risk genes revealed a consistent upregulation pattern (**Figures 6C–E**). Subsequently, we computed individual risk scores using the established methodology. We analyzed the correlations of risk genes or risk scores with lactylation across sepsis patients. The results demonstrated the evident positive correlations between lactylation level and risk scores or risk genes (**Figures 6F–I**). Consequently, the lactylation-related prognostic signature had a strong association with lactylation, which could predict the prognosis of sepsis from the perspective of lactylation.

In the following analyses, the temporal dynamics of risk gene expression were assessed within individual patients. The GSE95233 whole blood transcriptional profiling contained information for different time points. Some sepsis patients were sampled a second time at day 2 or day 3 since the admission day according to the clinical information. The transcriptional levels of the three risk genes, derived risk scores, and lactylation measurements were assessed across survival outcomes, respectively. The lines depicted in the plot between different time groups represented the same patients. Notably, in the survivor patients, both lactylation and the risk score showed a evident decrease at day2 or day3 (**Figure 6J**). Moreover, the risk genes PPP1R15A and FAM3A exhibited a similar tendency of decrease as the time progressed (**Figure 6J**). Unlike the survivors’ group, the level of lactylation and risk score maintained high levels during the progression, and the risk genes showed no decrease during the progression (**Figure 6K**). Thus, we considered that the persistent high level of lactylation might contribute to the high rate of mortality in the progression of sepsis.

### 3.7 *In vitro* experiments and clinical validation of the prognostic model in our own cohort

**Figure 7:**
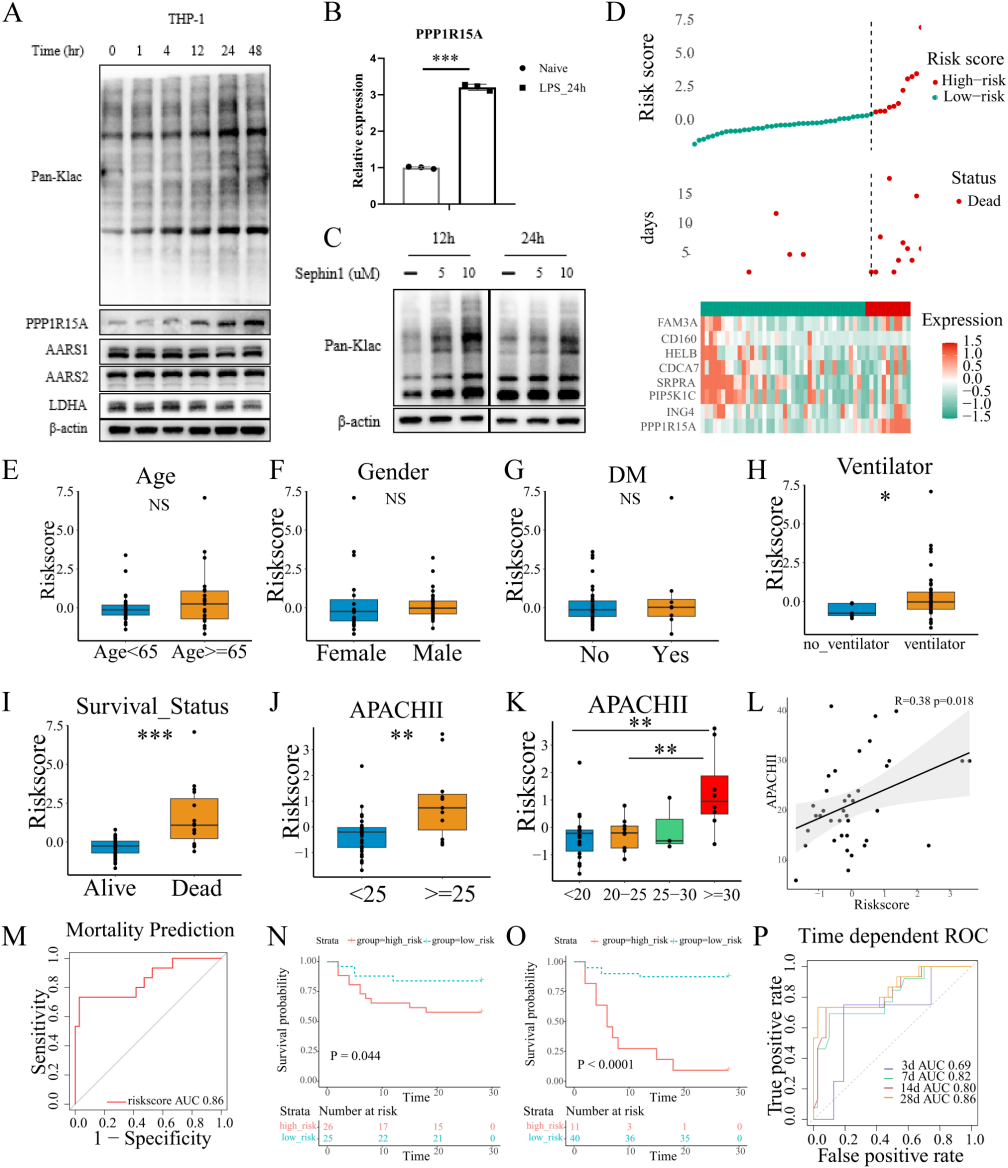
Validation of lactylation-related signature in our own cohort. **(A)** THP-1 cells were treated with LPS in a time-dependent manner and blotted for PPP1R15A, AARS1, AARS2, LDHA, and Pan-Klac. **(B)** THP-1 cells were treated with LPS for 24h. The PPP1R15A showed a significant increase in mRNA level with β-actin as a control. The statistical difference between naive and LPS groups was determined using unpaired Student’s t-test. ***p<0.001 **(C)**. Sephin1 (a selective PPP1R15A inhibitor) increased the global level of lactylation in THP-1 cells. **(D)** The distribution of risk-scores, survival time, expression patterns of signature genes in our own ICU cohort. **(E-G)** The risk-scores showed no difference between patients with different age, genders, and history of diabetes. The statistical difference between two groups was determined using unpaired Student’s t-test. P<0.05 was considered statistical significant [**(E-K)**, *p< 0.05, **p < 0.01, ***p < 0.001] **(H)**. The comparison of risk-scores between patients treated with ventilator or not in the first 24h after ICU admission. **(I)** The comparison of risk-scores between patients with different survival outcomes. **(J-L)** The correlation of risk-scores and APACHII scores. **(M)** The performance of the signature was evaluated with ROC curves in the prediction of mortality. **(N, O)** The Kaplan-Meier plot revealed different survival outcome between high and low risk groups [**(N)**, the median value of risk-scores of our own cohorts was set as the cut-off value, Log-rank test p=0.044. **(O)**, the optimal cut-off value was determined by X-title, Log-rank test p<0.0001]. **(P)** The performance of the signature was evaluated with ROC curves in the prediction of survival time.

To study the global lactylation in monocytes, THP-1 cells were subjected to lipopolysaccharide (LPS) for indicated times to trigger acute inflammation. Pan-Kla denotes broad detection of lysine lactylation modifications across the proteome, serving as a global indicator of cellular lactylation status. The cellular lactylation level was measured by Western Blot (anti-Pan-Klac). Accordingly, the expression of Pan-Klac and PPP1R15A were significantly increased in a time-dependent manner after LPS stimulation, with the results demonstrating clear statistical significance (**Figure 7A**; **Supplementary Figure S7**). Similarly, the mRNA level of PPP1R15A was significantly increased in THP-1 cells subjected to LPS for indicated times (**Figure 7B**). Thus, Sephin1, a selective PPP1R15A inhibitor, was used to explore the potential role of PPP1R15A in the global lactylation modification. Compared to the LPS stimulation alone, the LPS plus Sephin1 group showed a higher level of lactylation. Notably, GLUT1, a critical glucose transporter, exhibited similarly up-regulated expression levels following treatment with the PPP1R15A inhibitor. (**Figure 7C**; **Supplementary Figure S6A**). The above results suggest that PPP1R15A exerts significant effects on immune cell lactylation modification.

To further validate the predictive performance of the lactylation-derived prognostic signature, we collected the blood samples from the ICU at our institution (N=51) and isolated the PBMCs within the first 24 h of ICU admission. The relevant clinical information was detailed in **Supplementary Table S4**. Total RNA was extracted and reversed into cDNA. Then RT-qPCR was performed to quantify the relative expression of the eight genes (CD160, HELB, ING4, PIP5K1C, SRPRA, CDCA7, FAM3A, PPP1R15A) with β-actin as the control. The optimal risk score cutoff was identified using X-tile. As shown in the **Figure 7D**, the high-risk group showed an evident increase in the expression of PPP1R15A. Then we compared the risk scores among different clinical characteristics (including age, gender, the history of diabetes, chronic pulmonary disease, chronic heart disease, chronic renal disease, source of infection, CRRT utilization), and no statistically significant differences were observed (**Figures 7E–G**, **Supplementary Figures S8A–E**). However, patients receiving mechanical ventilation within 24 hours of admission showed a higher risk scores compared to those without ventilator support (**Figure 7H**). Notably, the risk scores were significantly higher in deceased patients compared to survivors (**Figure 7I**), which was in line with the analyses before. Additionally, the APACH II scores, which are widely used to evaluate the severity of ICU patients, showed a strong association with risk scores (**Figures 7J–L**, patients with high risk scores demonstrated elevated APACHE II scores). The signature demonstrated significant prognostic value, with ROC analysis confirming its robust predictive performance in our cohort (**Figures 7M–P**). For 28-day mortality prediction, the AUC reached 0.86 (**Figure 7M**). Time-dependent AUC values for survival prediction were 0.69 (3-day), 0.82 (7-day), 0.80 (14-day), and 0.86 (28-day) (**Figure 7N**). Notably, low-risk patients exhibited significantly improved survival outcomes compared to their high-risk counterparts (**Figures 7N–O**). Collectively, the lactylation-related prognostic signature exhibited a good performance and stability in our own cohort.

## 4 Discussion

Sepsis is a severe medical condition with a high rate of mortality, which has been defined as a global health issue (21). The dysregulation of the inflammatory responses is commonly observed in sepsis patients (22). The dynamic balance and imbalance between SIRS and CARS contribute to the high heterogeneity of sepsis (23). In recent decades, significant breakthroughs have been made in the pathogenesis of sepsis (24). Despite the advances in insights into the pathophysiology underlying the disorder, sepsis still lacks of reliable diagnostic and prognostic biomarkers (25), which drives the scientific researchers to explore the sepsis molecular mechanism from multiple aspects. Lactylation, which is a newly identified post-translational modification, has been demonstrated to play a role in multiple disease processes (11). It has been proved that glycolysis is significantly enhanced in sepsis (26), which results in the high concentration level of lactate, therefore increasing the global lactylation level. Lactylation plays important roles in multiple diseases including sepsis. Molecularly, lactylation enhances or weakens the original biological functions of certain molecules, or produces novel effects (27). In recent years, several studies have revealed that lactylation could regulate the activities of transcription factors, histones, mitochondrial-related proteins (28), thus having the potential to impact the balance of immune response or contribute to the immune paralysis in sepsis. However, the understanding of lactylation is not sufficient, and we lack efficient, simple, and affordable approaches to measure the level of lactylation in sepsis. To fill this gap, we sought to probe the dynamic changes of lactylation and establish a signature that could quickly and stably predict the mortality and survival outcome of sepsis patients. By utilizing integrative multiple analyses and *in vitro* investigations, we elucidated the potential molecular mechanisms mediating lactylation. In addition, lactylation, particularly histone lactylation, emerges as a key metabolic-epigenetic mechanism in trained immunity, as the lactate-lactylation axis directly couples glycolytic metabolism to long-term innate immune memory (29). These findings suggest that lactylation may also play a functional role in sepsis-induced trained immunity.

In our current study, by utilizing consensus clustering, four distinct lactylation-related sepsis subclusters were discovered. Then we depicted the characteristics and immune landscape of different subclusters using the bioinformatics analyses. The results revealed that the sepsis patients in cluster 3 had the highest level of lactylation. In addition, according to the immune-related calculation, the immunosuppressive effects were more pronounced in cluster 3 compared to the other clusters. Notably, as the Kaplan-Meier survival curves depicted, sepsis patients in cluster-3 had an evident poor survival outcome in comparison with the others. Thus, cluster 3 was defined as the special cluster, and from this, we sought to explore the hub genes involved in the lactylation using several bioinformatics analyses and machine learning. Briefly, we overlapped the genes that were altered both in cluster 3 and dead patients, and filtered out the variables without prognostic value. The candidate gene number was further reduced using LASSO and Elastic Net. Lastly, multivariate Cox regression identified critical hub genes, while pseudotime trajectory analysis revealed progressive increases in both risk gene expression and lactylation levels during sepsis progression. Notably, by separating the sepsis transcriptional profiling of survivors and non-survivors, we noticed that the lactylation level of non-survivors sustained a high degree, while lactylation in survivors showed the opposite, demonstrating that the lactylation might contribute to the mortality. While pseudotime reconstruction provided valuable insights into lactylation dynamics during sepsis progression, several methodological constraints warrant consideration: First, while our application of pseudotime analysis to bulk RNA-seq data is theoretically justified by the continuous development of sepsis progression, we acknowledge the approach was originally developed for single-cell transcriptomics. Second, the approach cannot resolve dynamics at single cell level, potentially masking cell-type-specific patterns. These limitations highlight the need for future validation using single-cell RNA-seq in longitudinal cohorts.

Besides, we established a sepsis lactylation-related signature utilizing the hub genes explored in above analyses. The signature had a good and stable performance in the prediction of survival time and mortality outcome in sepsis patients. Specifically, in the training set, the AUC values of the signature were 0.77, 0.77, and 0.78 at 3, 14, and 28 days, respectively. The AUC value of mortality prediction reached 0.78. In the validation cohort, the AUC values of the signature were 0.73, 0.70, and 0.73 at 3, 14, and 28 days, and the AUC value of mortality prediction reached 0.73. The signature and risk genes were further validated in an external whole blood transcriptional profiling and a single-cell RNA-sequencing of human PBMCs. Overall, the lactylation-related signature showed a good performance and high accuracy. Moreover, to increase the credibility of the results, we sampled the sepsis blood in our own hospital and isolated the RNA at intensive care unit admission. The expression level of genes involved in the signature were quantified using rt-qPCR. By integrating the PCR results and the corresponding clinical information of sepsis patients, we further demonstrated that the lactylation-related signature has good performance.

Additionally, PPP1R15A (GADD34) which could prevent the hyperphosphorylation of the translation initiation factor eIF-2α (30), has been identified as the hub gene with high risk coefficient. Endoplasmic reticulum (ER) stress represents a pathological state induced by excessive unfolded/misfolded protein accumulation in the ER (31), often occurs in cancer and infectious diseases (32). PERK (Protein kinase RNA-like Endoplasmic Reticulum Kinase) undergoes autophosphorylation, which induces the phosphorylation of the eukaryotic translation initiation factor 2α. It serves a dual role: it suppresses overall protein synthesis to alleviate the burden of nascent proteins on the ER, while selectively enhancing the translation of certain molecules, including activating transcription factor 4 (ATF4). ATF4 subsequently increases the mRNA level of PPP1R15A (28). A recent study suggests that ATF4 could increase the expression level of GLUT1, subsequently allowing glucose greater access to the intracellular environment, resulting in a high global glycolysis level (33). The similar phenomenon was confirmed in our *in vitro* experiment. Moreover, Sephin1, a selective PPP1R15A inhibitor (34), has been used to explore the potential effects of PPP1R15A. Consistent with our preconceptions, the expression level of PPP1R15A could indicate the lactylation, and the activity of PPP1R15A could alter the global lactylation level.

In conclusion, we have demonstrated the dynamic changes of lactylation in sepsis through a comprehensive integrated analysis. This study establishes an innovative approach for prognosis of sepsis patients, but also a novel insight into the pathophysiology mechanism of sepsis from the lactylation perspective. The following limitations of this research should be noted: Firstly, not many comparisons have been performed in the characteristics analysis due to the limited clinical information. Certain cohorts were missing data on outcomes and survival time, which is unavoidable since it was a retrospective analysis. In our study, though we directly measured the overall lactylation of the THP-1 cells, the GSVA-based lactylation scores was served as inferences for cellular lactylation activity in the clinical cohorts, which is not a direct post-translational modification measurement. Thus, our findings demonstrate that while the computationally inferred values based on related gene expression patterns in the transcriptome might serve as indicators of lactylation activity, they do not fully represent the comprehensive lactylation status at the cellular level. Considering the limitation, future lactylation proteomics analyses and studies are needed for definitive quantification. Despite utilizing clinical samples and analyzing multiple datasets in this study, the findings require validation in larger, independent clinical cohorts to determine their practical clinical applicability.

## 5 Conclusions

This study systematically investigated the lactylation in sepsis, revealing its significant association with disease progression and patient outcomes. Through comprehensive bioinformatics analyses of multiple datasets, we identified four distinct lactylation-related sepsis subclusters. We further developed an 8-gene lactylation-based prognostic signature (CD160, HELB, ING4, PIP5K1C, SRPRA, CDCA7, FAM3A, PPP1R15A) that effectively predicted mortality risk in both training (AUC 0.77-0.78) and validation cohorts (AUC 0.70-0.73). *In vitro* experiments confirmed that PPP1R15A inhibition altered global lactylation levels. Clinical validation in our own hospital using patient-derived PBMCs supported the signature’s predictive accuracy (28-day mortality AUC=0.86). Based on the dysregulated immune cell metabolism, a novel signature was established with potential applicability for rapid prognosis prediction in sepsis management.

## Data Availability

Publicly available datasets were analyzed in this study. This data can be found here: Publicly available datasets were derived from GEO (accession number GSE65682, GSE95233, GSE167363).
