# Supplementary material for: A novel, rapid, and practical prognostic model for sepsis patients based on dysregulated immune cell lactylation
Source: Front Immunol. 2025 Jun 19;16:1625311. doi: 10.3389/fimmu.2025.1625311 (PMC12221935; doi:10.3389/fimmu.2025.1625311)
Supplement: Supplementary file 1 [file DataSheet1.pdf]

Fig.S1

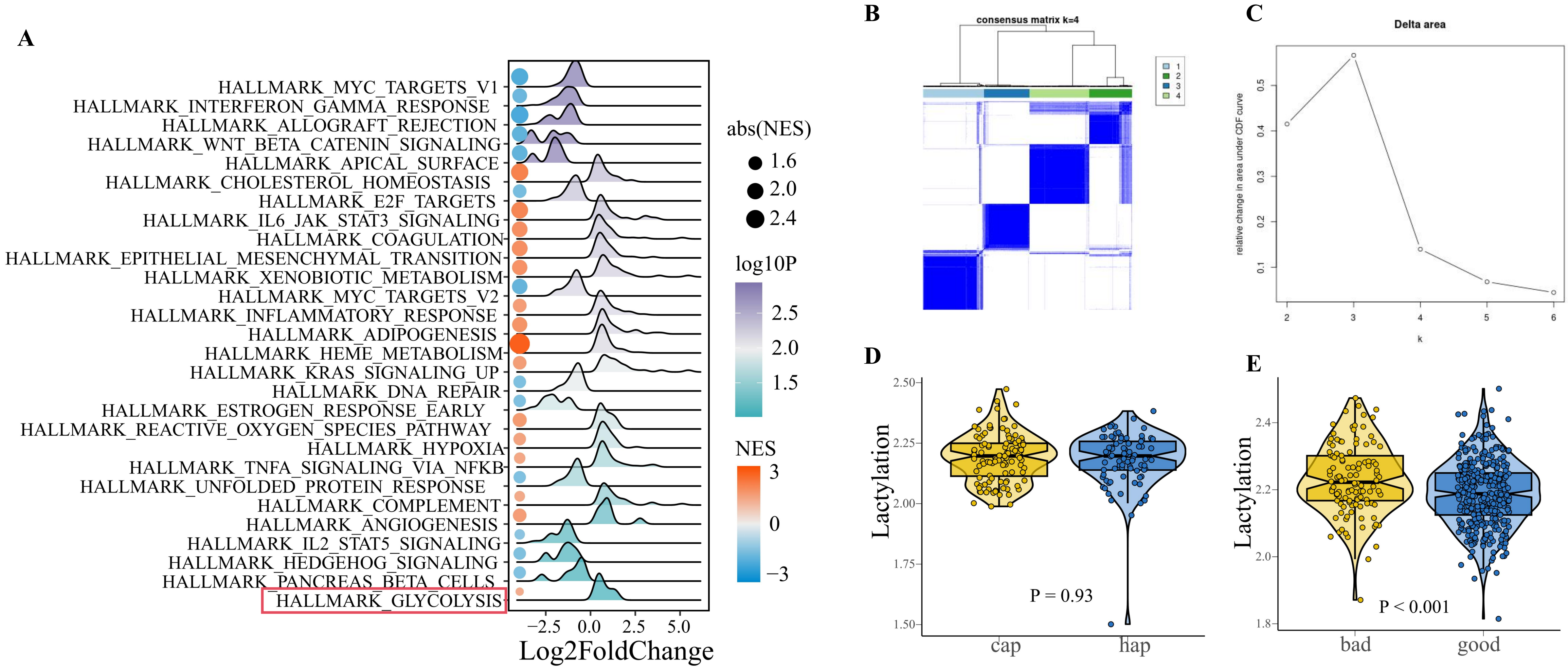

Fig.S1 Consensus clustering of sepsis patients based on lactylation-related genes. A. Gene Set Enrichment Analysis (GSEA) showed various biological processes enriched in sepsis patients, including Glycolysis. B-C. The optimal clustering K value selection according to the CDF curve. D. The comparison of lactylation between CAP and HAP showed no difference. E. The comparison of lactylation between bad and good status patients according to the clinical information. The lactylation level was higher in bad status patients.

Fig.S2

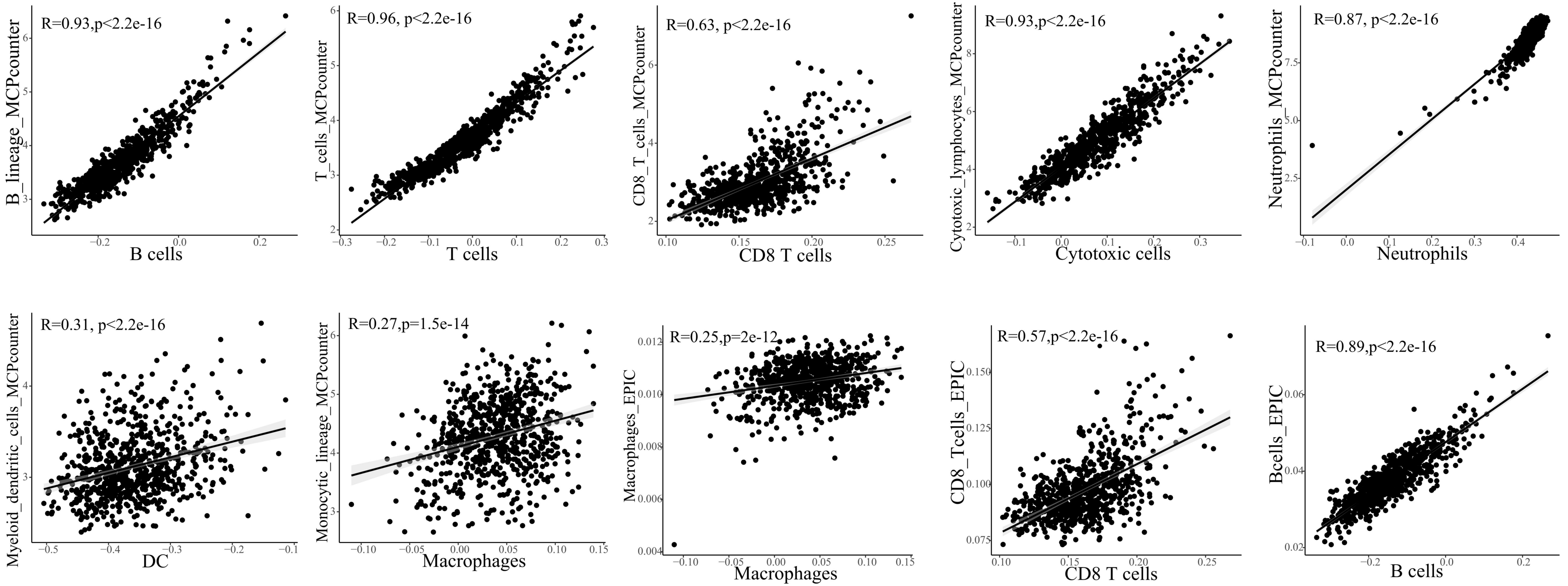

Fig.S2 Correlation of immune cells calculated by different algorithms. Pearson analysis of immune cells calculated by ssGSEA, EPIC, or MCPcounter.

Fig.S3

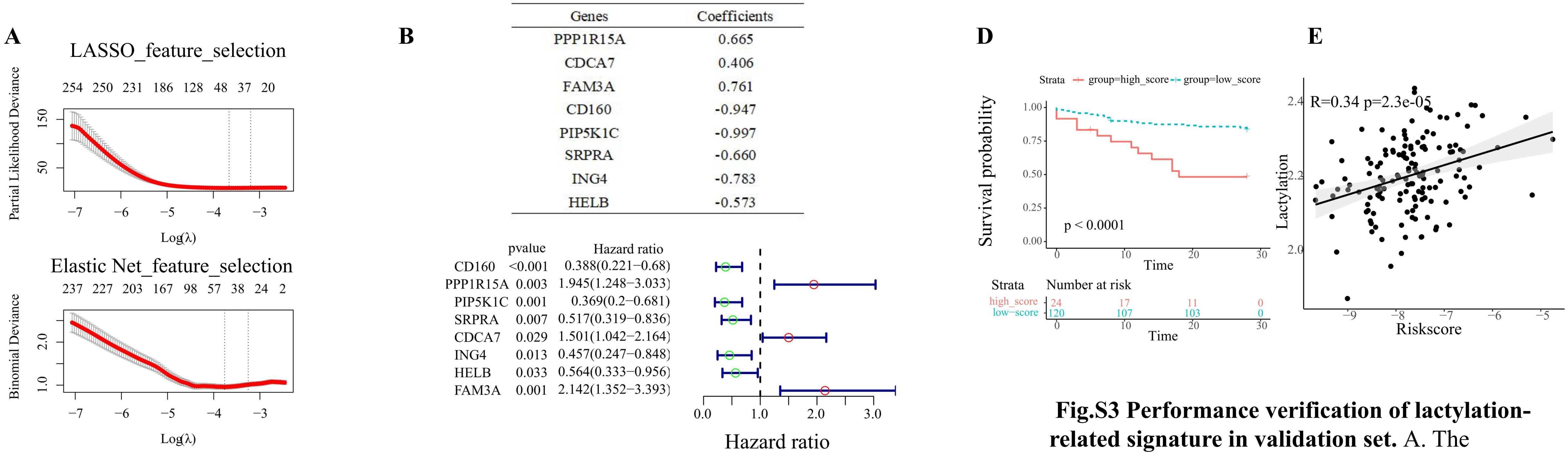

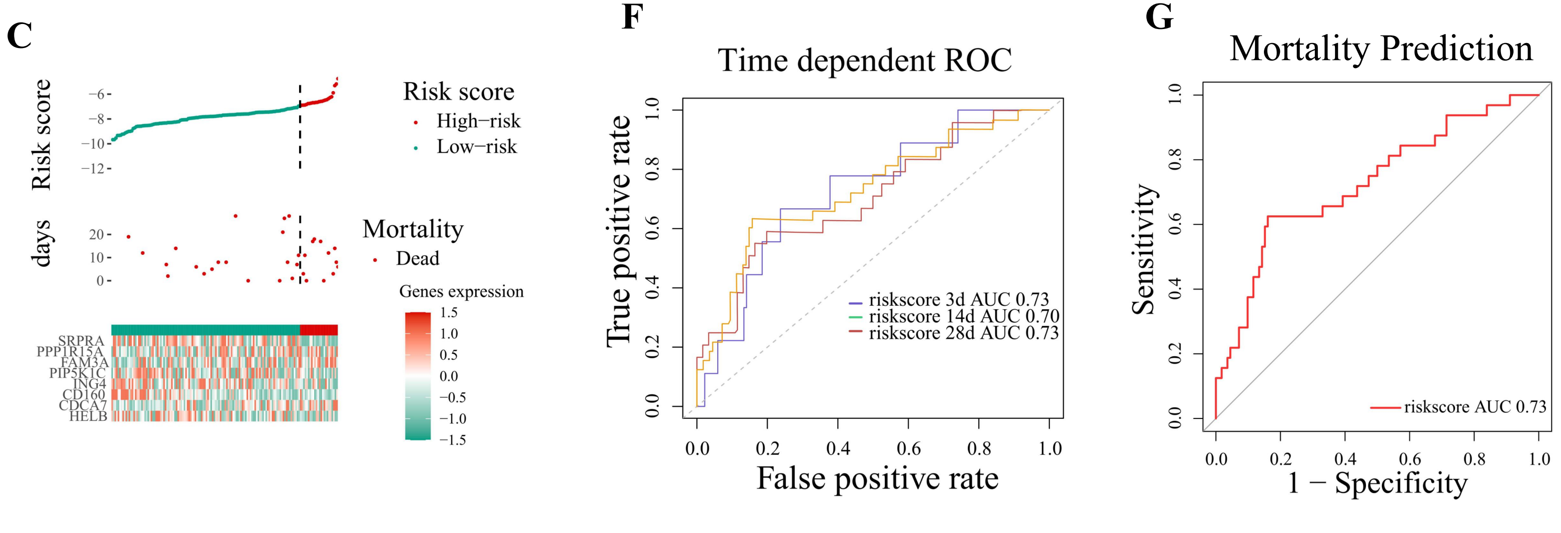

Fig.S3 Performance verification of lactylation-related signature in validation set. A. The variable selection process via LASSO regression and Elastic Net. B. The regression coefficients table and hazard ratios (HR) of the 8 hub genes. C.The distribution of risk scores, survival time, expression patterns of signature genes in the validation set. D. Kaplan-Meier plot revealed the different survival outcome between patients in high and low risk groups. E. Pearson analysis of lactylation and risk scores in the validation set. F-G. The ROC curves confirmed the signature performance of in the prediction of survival time and mortality in the validation set.

Fig.S4

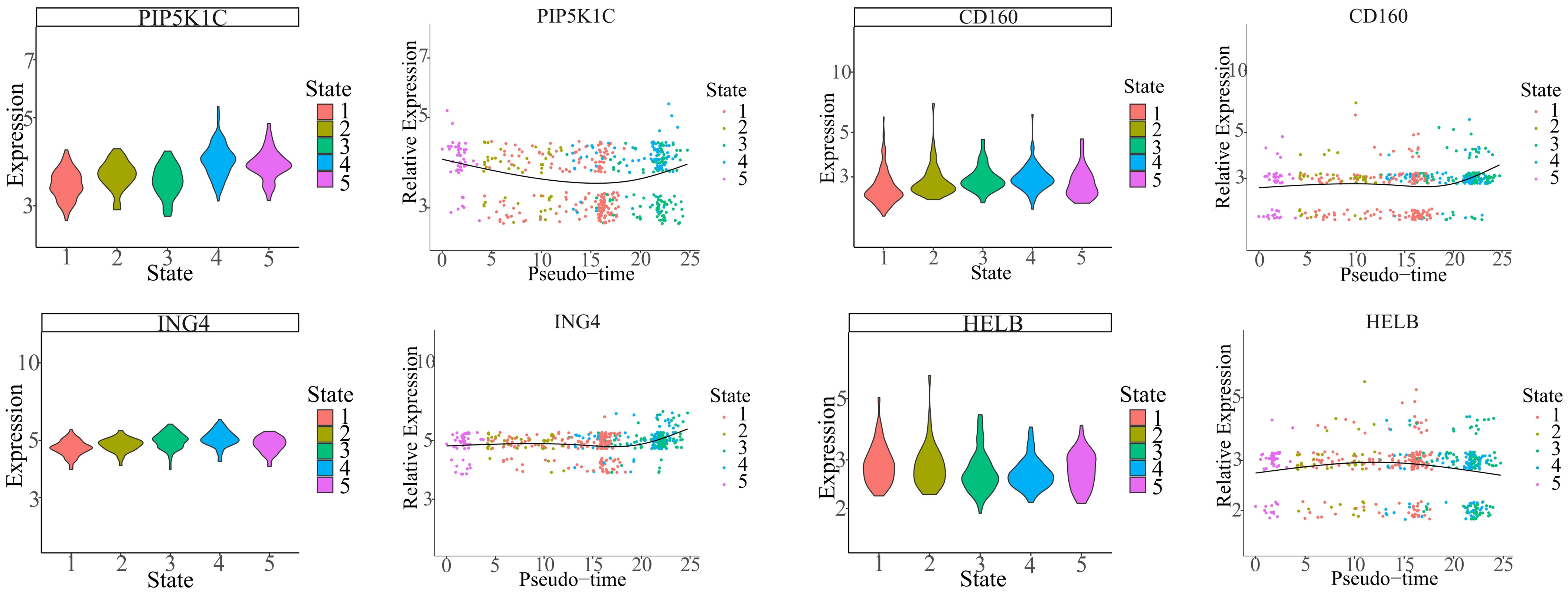

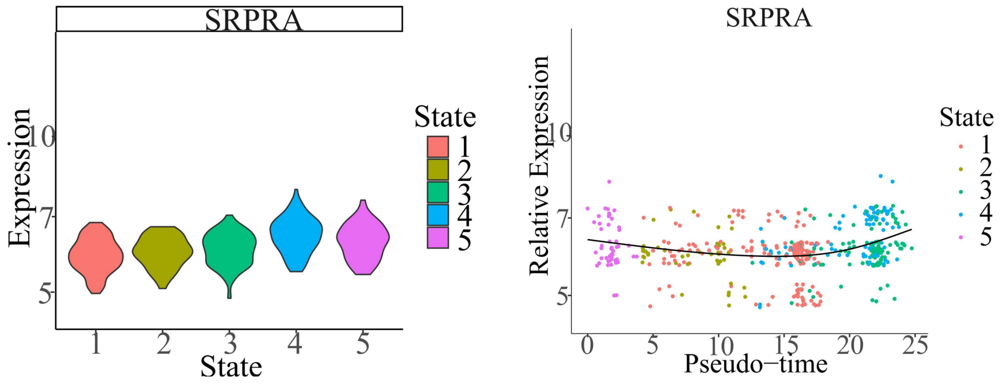

Fig.S4 Pseudo-time trajectories showed the expression level of hub genes (HR<1) changed with the enrichment level of lactylation.

Fig.S5

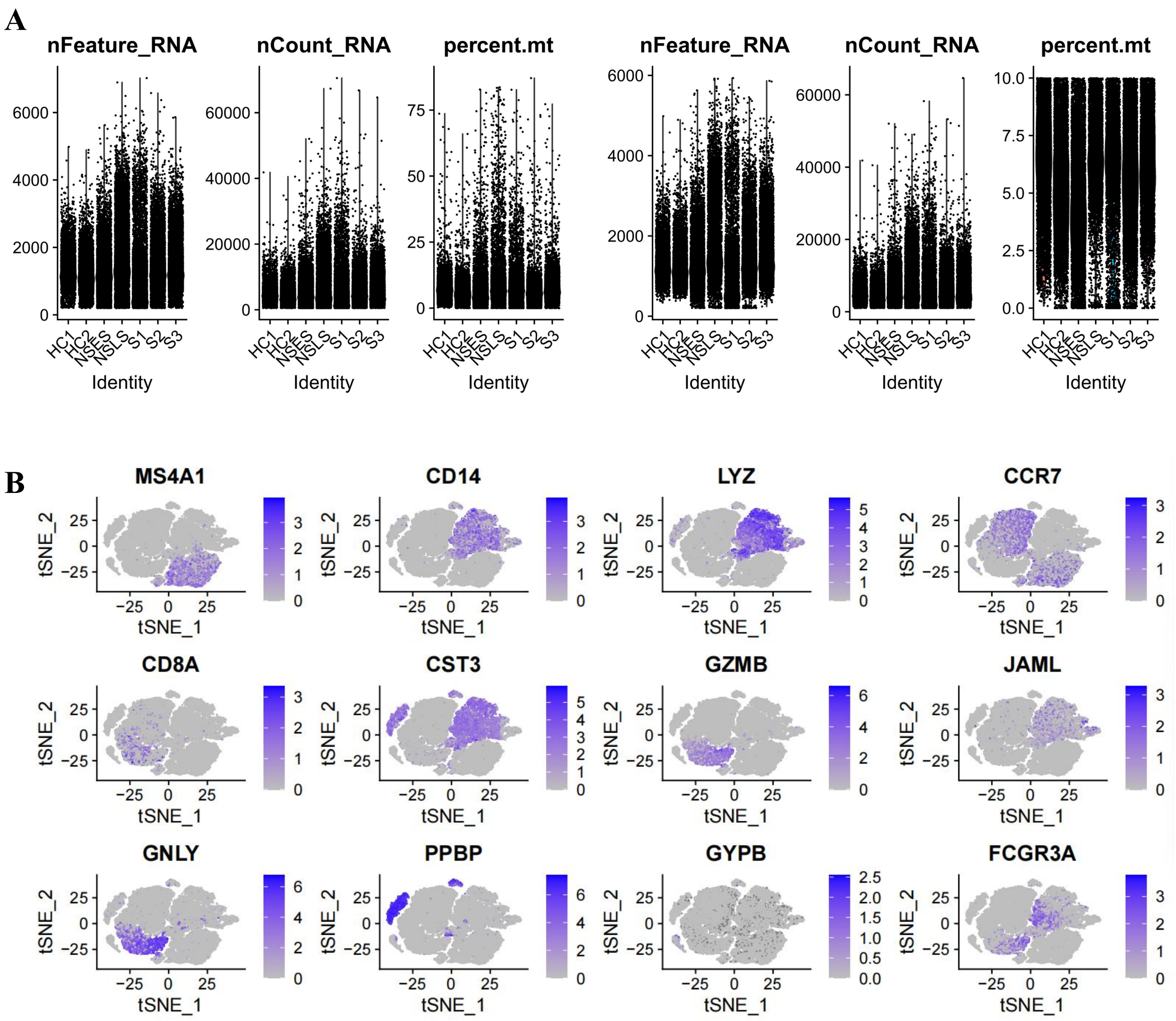

Fig.S5 Quality controls of PBMC single-cell RNA sequencing data in GSE167363. A. Cells with low quality were filtered out according to "nFeature_RNA", "nCount_RNA", and "Percent of mitchondrial". B. Identfication of immune cell clusters with canonical markers annotated in the t-sne plot. C. Different cell proportion of healthy volunteers, survivors, and non-survivors.

Fig.S6

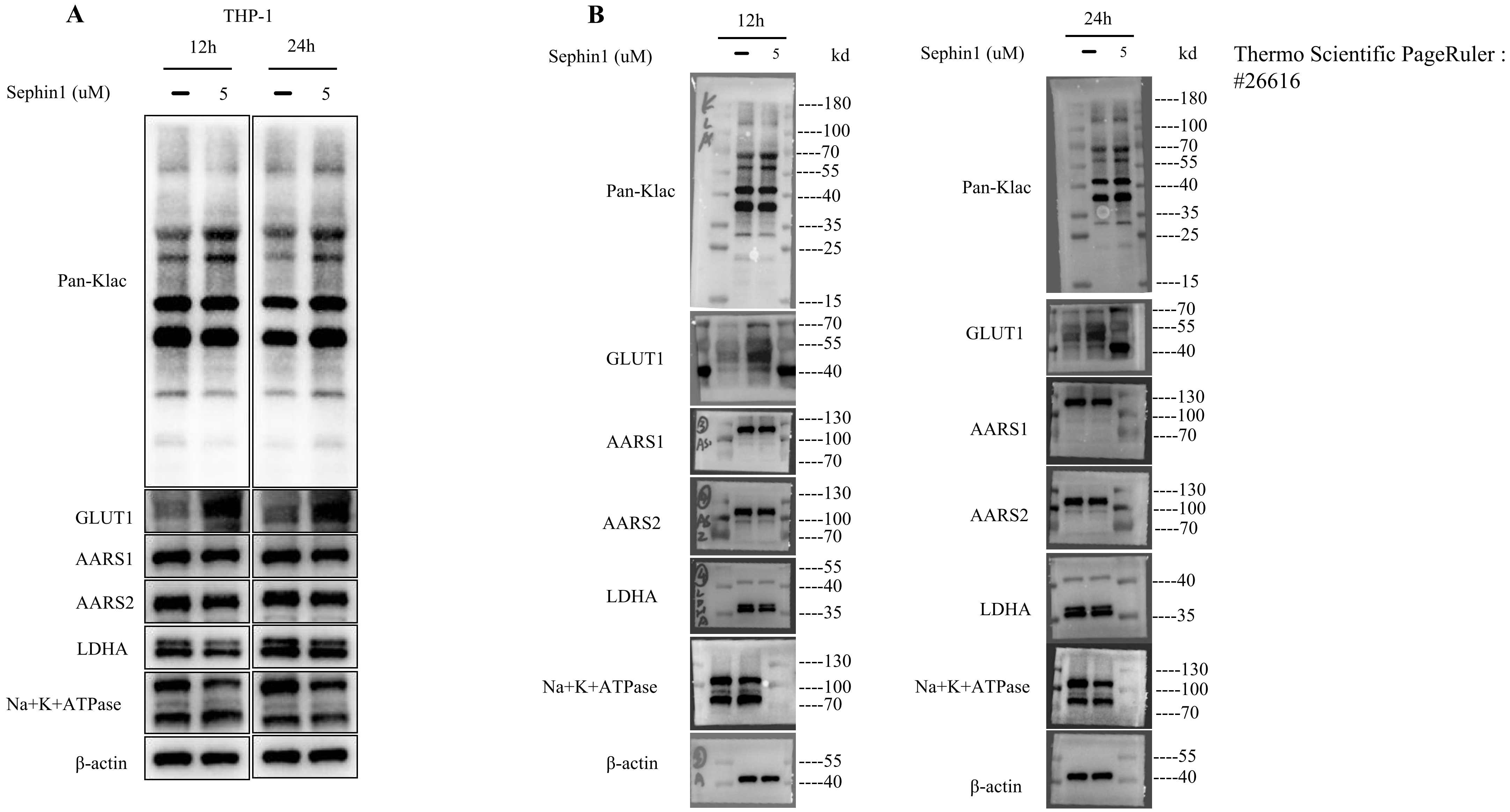

Fig.S6 Inhibition of PPP1R15A increased the Pan-Klac level. A. The protein (Pan-Klac, GLUT1, AARS1, AARS2, LDHA) changes with PPP1R15A inhibitor (Sephin1) and LPS stimulation for indicated time. B. Raw images for Western blot data in Fig.S6A

Fig.S7

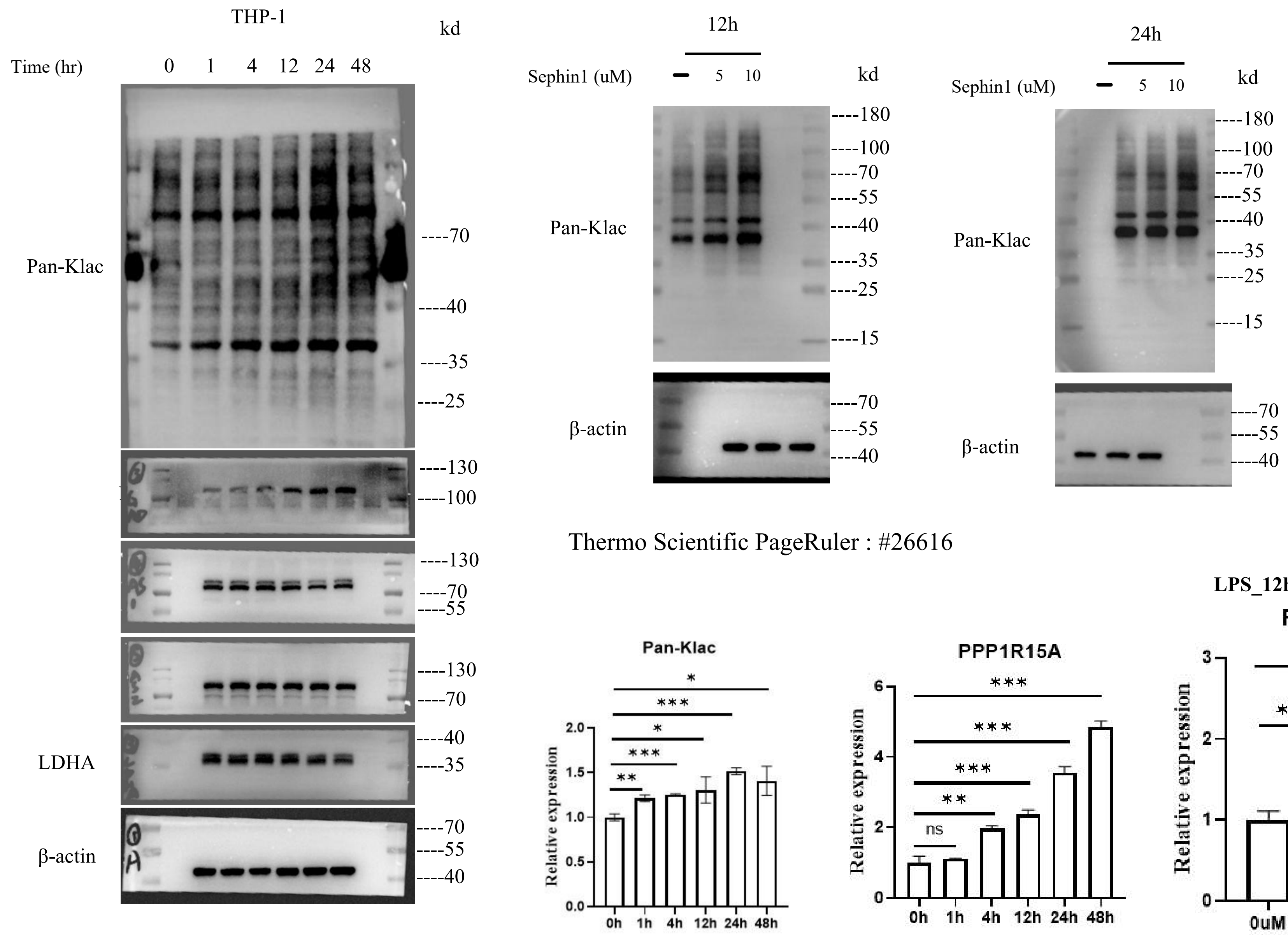

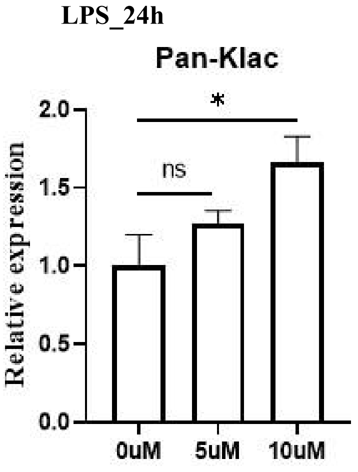

Fig.S7 RAW image and statistical results (Comparison of different time points with 0h. Comparison of different concentrations of Sephin1 with 0uM) for the Western blot data in Figure 7.

Fig.S8

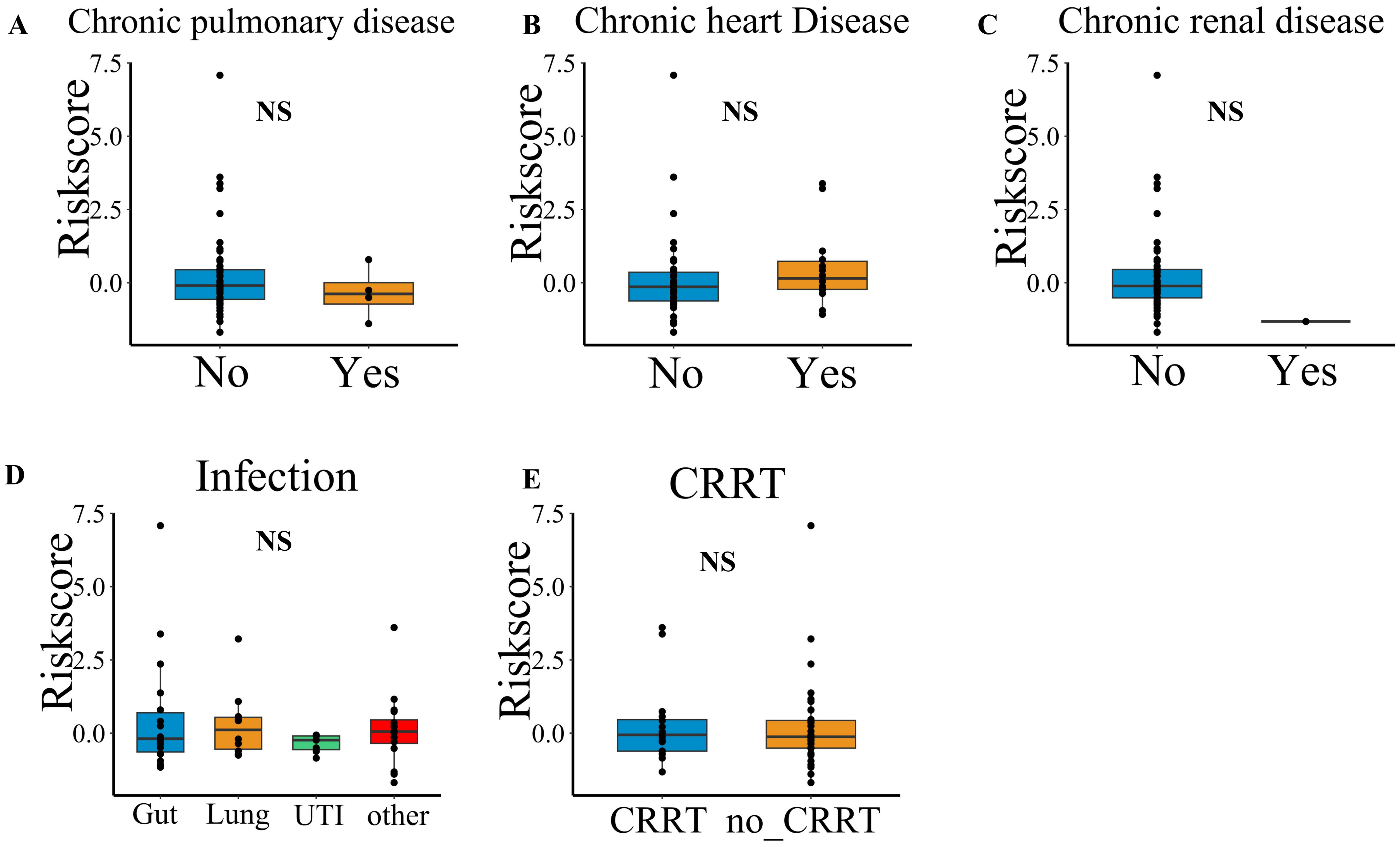

Fig.S8 The correlation between riskscore and clinical characteristics
