# Supplementary material for: A novel, rapid, and practical prognostic model for sepsis patients based on dysregulated immune cell lactylation
Source: Front Immunol. 2025 Jun 19;16:1625311. doi: 10.3389/fimmu.2025.1625311 (PMC12221935; doi:10.3389/fimmu.2025.1625311)
Supplement: Supplementary file 3 [file Table2.docx]

| Cells | Markers |
| --- | --- |
| B cells | MS4A1 |
| T cells | CD3 |
| erythroblast | GYPB, AHSP |
| Monocytes | CD14 |
| DCs | FCER1A, CST3 |
| neutrophils | JAML, SERPINB |
| NK cells | GNLY, NKG7 |
| platelets | PPBP |

**Table.S2 The canonical markers for different immune cells.**
