# Supplementary material for: A novel, rapid, and practical prognostic model for sepsis patients based on dysregulated immune cell lactylation
Source: Front Immunol. 2025 Jun 19;16:1625311. doi: 10.3389/fimmu.2025.1625311 (PMC12221935; doi:10.3389/fimmu.2025.1625311)
Supplement: Supplementary file 4 [file Table3.docx]

| Gene | Sequence (5’-3’) |
| --- | --- |
| CD160 | F: ATGTTCACCATAAGCCAAGT |
|  | R: TTTGTTTCAATCCCGTCA |
| PPP1R15A | F: AACCTCTACTTCTGCCTTGTC |
|  | R: CTCCGTGGCTTGATTCTC |
| PIP5K1C | F: ACCAGCGACGACGAGTTCA |
|  | R: ACCACGACGCGGATGTTC |
| SRPRA | F: CCTTGGCTGACCATTCTA |
|  | R: GGTGCCCACAAAGACGAT |
| CDCA7 | F: GGCTCCGACTCACAATCA |
|  | R: TCCACGGTCTTCCTCTTT |
| ING4 | F: AAAACTCGGATGAAGAAGC |
|  | R: AGGCAATAGGTGGGTTCG |
| HELB | F: GCCAGTTCTCAGTCATCT |
|  | R: ATCACAGTCTTGCCTTCT |
| FAM3A | F: GCTGAACATCGCCCTGGTG |
|  | R: TGGTGGCTGGGTCGTCGTA |
| β-actin | F: TTGAAGGCTGGATTTCCTTTGGGC |
|  | R: TCGTCGCAGATGAAATAGGGCTGT |

**Table.S3 The primer sequences of genes involved in the signature.**
